# A novel protein-preserving passive tissue clearing approach using sodium cholate and urea for whole-organ imaging

**DOI:** 10.1038/s12276-025-01550-w

**Published:** 2025-10-01

**Authors:** Kitae Kim, Kwangbae Lee, Taehoon Kang, Jungmihn Lee, Wonseok Lee, Ji Yeoun Lee, Sunghoe Chang

**Affiliations:** 1 Department of Physiology and Biomedical Sciences Seoul National University College of Medicine, Seoul, South Korea; 2 Department of Anatomy and Cell Biology Seoul National University College of Medicine, Seoul, South Korea; 3 Department of Transitional Medicine Seoul National University College of Medicine, Seoul, South Korea; 4 Department of Neurosurgery Seoul National University College of Medicine, Seoul, South Korea; 5 Division of Pediatric Neurosurgery, Seoul National University Children’s Hospital, Seoul, South Korea; 6 Neuroscience Research Institute, Seoul National University College of Medicine, Seoul, South Korea

**Keywords:** Tissue engineering, Histology

## Abstract

The recent advancements in tissue-clearing techniques have opened new possibilities for non-invasive three-dimensional (3D) volumetric imaging of a wide range of biological specimens. Passive tissue-clearing methods use diffusion-based processes to infiltrate clearing reagents into samples without mechanical forces or energy input, aiming to minimize the sample disruption while preserving the tissue architecture and molecular information. Nevertheless, these methods often rely on sodium dodecyl sulfate (SDS) as a delipidating detergent, which has a risk of causing tissue damage and protein disruptions, thus necessitating the development of a reliable yet accessible approach for passive tissue clearing. Here we replaced SDS with sodium cholate (SC), combined it with urea and developed OptiMuS-prime as a novel passive tissue clearing technique to achieve a better passive infiltration of clearing reagents while retaining structural integrity. SC, a non-denaturing detergent with small micelles, enhances tissue transparency while preserving proteins in their native state, whereas urea disrupts hydrogen bonds and induces hyperhydration to enhance probe penetration. Through the optimization of composition and protocols, we found that OptiMuS-prime enables the 3D imaging of immunolabeled neural structures and vasculature networks across multiple rodent organs, including the brain, intestine and lung. The method demonstrated robust clearing and immunostaining capabilities, particularly for detecting subcellular structures in densely packed organs such as the kidney, spleen and heart, as well as in post-mortem human tissues and human induced pluripotent stem cell-derived brain organoids. Together, OptiMuS-prime offers a fully accessible and customizable solution for passive clearing and immunostaining, enabling 3D cellular connectivity analysis across whole organisms without the need for extensive tissue-clearing expertise.

## New passive tissue clearing method enhances three-dimensional imaging

Recent advances in tissue clearing techniques have revolutionized the three-dimensional (3D) imaging of biological tissues. This Article study introduces a new method called OptiMuS-prime. OptiMuS-prime combines sodium cholate, a bile salt detergent, with urea to enhance tissue transparency while preserving the structure and protein integrity. The Article involved experiments on mice and human tissues, using a passive clearing approach that does not require specialized equipment. This method effectively clears and stains tissues, allowing the detailed 3D imaging of blood vessels and neural networks. The results show that OptiMuS-prime provides superior tissue clearing compared with other methods, while maintaining original tissue size and fluorescence signals. The technique proves particularly effective for densely packed tissues, such as post-mortem human samples and brain organoids. In conclusion, OptiMuS-prime offers a reliable and accessible solution for 3D imaging in biological research.

This summary was initially drafted using artificial intelligence, then revised and fact-checked by the author.

## Introduction

The recent advancements in various tissue clearing techniques have ushered in a new era of section-free three-dimensional (3D) deep tissue imaging^1–3^. These methods are typically categorized into organic solvent-based clearing or aqueous-based clearing methods^4–6^. Hydrophobic tissue clearing, utilizing organic solvents, enables long-term specimen preservation, facilitating multiple imaging sessions. However, the dehydration step in this method tends to diminish fluorescent signals and reduce tissue size^7–9^. Conversely, hydrophilic tissue clearing uses water-soluble reagents for refractive index (RI) matching. Although hydrophilic tissue clearing may exhibit lower optical transparency than hydrophobic clearing, it provides benefits in terms of biosafety and preservation of fluorescence signals^10–12^.

Unlike active tissue clearing methods, such as CLARITY^13^, passive clearing methods rely on diffusion-based processes to introduce clearing agents into tissue samples. Although slower, these methods are less likely to cause structural damage compared with active methods, thereby preserving the tissue architecture and molecular information. Furthermore, passive clearing methods do not require specialized equipment, offering a straightforward and accessible approach for researchers without expertise in tissue clearing. However, previous passive clearing methods often used sodium dodecyl sulfate (SDS) as a delipidation detergent^13–16^. Despite its effective delipidation, SDS’s high aggregation number (80–90) and low critical micelle concentration (CMC, 8 mM) result in large micelles that are difficult to wash out^17–20^. Moreover, SDS’s potent detergent properties have a risk of causing tissue deformation and protein disruption^21–23^.

Addressing these limitations of SDS, we previously developed an active clearing method named SCARF by utilizing sodium cholate (SC) as a detergent instead of SDS. SC, a bile salt detergent with a steroidal structure featuring a hydrophobic convex side and a hydrophilic concave side, exhibits facial amphiphilicity, a higher CMC (14 mM) and a lower aggregation number (4–16) and forms substantially smaller micelles compared with SDS^24–27^. We reported that due to the superior properties of SC, SCARF renders tissue transparency orders of magnitude faster than SDS-based methods while excellently preserving endogenous fluorescence and tissue integrity and enabling more efficient antibody penetration^17^.

We also developed OptiMuS, a water-based optical clearing method designed to effectively enhance tissue transparency by matching RI^28^. By combining iohexol with urea’s hyperhydration capability to reduce light scattering and improve tissue penetration, and ᴅ-sorbitol’s gentle clearing and sample preservation capability, OptiMuS provides simple, fast and versatile optical clearing for the 3D imaging of various biological structures while preserving the size and fluorescent signal of the tissues. Nonetheless, as a simple RI matching solution without a delipidation capability, OptiMuS presents challenges for immunostaining deep biological tissues, thereby limiting its broader application.

Based on our results from SCARF and OptiMuS, we hypothesized that SC’s superior performance in tissue clearing compared with SDS, when combined with urea, would enhance passive tissue clearing efficacy without causing tissue damage or protein disruption. Here, we developed optimized method combining urea and SC for passive clearing and immunostaining effective (OptiMuS-prime), which utilizes a high concentration of urea to break hydrogen bonds and induce hyperhydration, critically balanced with SC detergent to achieve optimal tissue transparency and probe penetration while preserving protein and tissue integrity. Notably, SC alone in a urea-free solution was less effective at clearing and immunostaining compared with OptiMuS-prime (Supplementary Fig. 2). The method enables a robust clearing and immunostaining across analyzed tissues, with a particular advantage in effectively penetrating and clearing densely packed organs such as the kidney, spleen and heart and detecting subcellular structures in post-mortem human tissues and human brain organoids. OptiMuS-prime provides a fully accessible and customizable solution for passive clearing, immunolabeling and RI matching, requiring no specialized equipment or expertise in tissue clearing, thus making it ideal for both 3D volumetric studies and routine immunohistochemistry.

## Materials and methods

### Animals and human samples

The experiments utilized 30 male mice (C57BL/6N, aged 6–8 weeks) and six female Sprague–Dawley rats (aged 3 weeks) from KOATECH. The brain samples from Thy1-EYFP H-line transgenic male mice (*n* = 3, aged 6 months) were provided by Dr. Chang Man Ha of the Korea Brain Research Institute; the brain samples from ChAT-IRES-Cre::tdTomato male mice (*n* = 3, aged 3–4 weeks) were provided by Shin Hwa Yun of the Seoul National University College of Medicine. Animal husbandry and all experimental procedures involving mice were approved by the Institute of Animal Care and Use Committee guidelines of Seoul National University (SNU-220525-4-3). This study, which involved human participants, was reviewed and approved by the institutional review board at Seoul National University Hospital (Institutional Review Board E-2408-099-1562). According to national laws and institutional guidelines, written consent from participants was not required. In addition, the study did not include any images or data that could identify individuals.

### Sample preparation

For mice brain sample preparation, mice were deeply anesthetized with a mixture of zoletil (30 mg/kg) and rompun (xylazine; 10 mg/kg). Subsequently, the animals were transcardially perfused with 50 ml of phosphate-buffered saline (PBS), followed by 50 ml of 4% paraformaldehyde (PFA) (P6148, Sigma) using a peristaltic pump (MP-100, Tokyo Rikakikai) set to a flow rate of 7 ml/min. Following perfusion, all collected samples were postfixed by immersion in 50 ml of 4% PFA at 4 °C overnight, then rinsed with PBS before clearing. Brain samples with thicknesses of 1 mm and 3.5 mm were sectioned using a vibratome (VT1200s, Leica Biosystems). For post-mortem human brain and colon tissues, samples were incubated in 25% (v/v) *N*-methyldiethanolamine (471828, Sigma) in PBS at 37 °C for 12 h with shaking to remove the heme structures as a decolorization step.

### Brain organoids

The brain organoids were derived from the human induced pluripotent stem cell line no. 5-1 and cultured under feeder-free and mycoplasma-free conditions. The organoids were generated following protocols from previous reports^29,30^. In brief, both protocols involved key stages, including the formation of 3D embryoid bodies, neural induction, patterning and differentiation and maintenance. The initial 3D embryoid bodies were generated to establish a foundation for neural tissue, followed by neural induction using growth factors and signaling inhibitors. During patterning and differentiation, additional growth factors were applied to promote the development of neural structures. Finally, the organoids were maintained with medium changes every 3–4 days to support the maturation and long-term culture. The imaging was conducted on day 16 (D16) and D50 organoids developed using the Pasca method^30^ and on D21 organoids created using the Lancaster method^29^ to capture the distinct developmental stages of the organoids.

### Preparation of OptiMuS-prime

Tris-(hydroxymethyl)-aminomethane (Tris)–ethylenediaminetetraacetic acid (EDTA) solution was prepared by dissolving 100 mM of Tris (252859, Sigma) and 0.34 mM of EDTA (17385-0401, Junsei Chemical) in distilled water and adjusting the pH to 7.5. Subsequently, 10% (w/v) SC (C2154, Sigma), 10% (w/v) ᴅ-sorbitol (S7547, Sigma) and 4 M urea (29700, Thermo Scientific) were dissolved in the Tris–EDTA solution until completely dissolved at 60 °C. The solution was then cooled to room temperature (RT) and stored at RT for future use. The RI-matching solution (OptiMuS RI of 1.47) was prepared using the same procedure but replacing 10% (w/v) SC with 75% (w/v) Histodenz (iohexol; D2158, Sigma). This solution was stored at 4 °C for future use. All clearing procedures were conducted at 37 °C.

### OptiMuS-prime clearing

The fixed samples were immersed in 10–20 ml of OptiMuS-prime solution and placed in a 37 °C incubator with gentle shaking. The time required for clearing depends on the tissue type and thickness (150-µm-thick mouse brain: 2 min; 300–500-µm-thick mouse brain: 6 h; 1-mm-thick mouse brain: 18 h; 3.5-mm-thick mouse brain block, human brain organoid (D16 and D21) and whole mouse heart, lung and half kidney: 2–3 days; whole mouse brain, human brain organoid (D50), 3–5-mm-thick human brain blocks and human colon: 4–5 days; whole rat brain: 7 days). All clearing processes were performed in a 37 °C incubator (a 60 °C incubator for faster clearing is optional), and changing the solution once a day can improve the clearing efficiency.

### Immunostaining

For staining mouse brain slices, samples were first rinsed in PBS for 1 h and incubated in a permeabilization solution (2% (v/v) Triton X-100 (T8787, Sigma) in PBS) for 1 h at 37 °C in an incubator with gentle shaking. The samples were washed in PBS and incubated in a blocking solution (10% (w/v) bovine serum albumin (BSA) (BSAS 0.1, Bovostar) in PBS) for 1 h at 37 °C. The samples were then immunostained with primary antibodies and dye-conjugated secondary antibodies in a staining solution (3% (w/v) BSA and 2% (v/v) Triton X-100 in PBS) for 2 days each at 37 °C. The samples were washed in PBS for 10 min and immersed in OptiMuS solution for 1 h at 37 °C before the imaging. For staining whole mouse brains, whole rat brains, mouse organs, human tissues and human brain organoids, samples were incubated in permeabilization solution for 12 h with gentle shaking and washed for 1 h, followed by a blocking solution for 12 h at 37 °C. The samples were immunostained with primary antibodies for 4–5 days 37 °C followed by washing for 1–2 h and staining with secondary antibodies for 3–4 days at 37 °C. For whole rat brains, samples were stained with anti-GFAP-Alexa Fluor 488 conjugated antibody for 10 days at 37 °C. Human tissue specimens and brain organoids were processed following the same protocol used for mouse tissues, with modifications to the staining solution (2% (w/v) SC and 3% (w/v) BSA in PBS). The staining solution containing antibodies was replaced every 48 h to maintain optimal labeling efficiency. Following staining, all samples were washed in PBS for 6 h at RT and then immersed in OptiMuS for 1 day at 37 °C before imaging. All antibodies and staining probes used in the procedure are listed in Supplementary Tables 1 and 2 (Supplementary Information).

### Imaging

#### Confocal fluorescence microscopy

1. Samples (500-µm-thick mouse sample, a 1-mm-thick mouse brain and intestine and a 1-mm-thick human brain) were immersed in OptiMuS for 1 h at 37 °C for RI matching. The samples were kept in OptiMuS during imaging. The imaging was performed using an upright confocal microscope (C2Si, Nikon) with a Plan-Apochromat 10× objective lens (numerical aperture (NA) 0.5 and working distance (WD) 5.5 mm), and image acquisition was conducted using NIS-Elements software (Nikon). For the 3D reconstruction and rendering, IMARIS 9.6 (Bitplane AG) software was used.
2. The fluorescence imaging of brain sections (300-µm-thick mouse brain sample) was conducted using an inverted laser-scanning confocal fluorescence microscope (LSM980, Zeiss). The samples were placed on a slide glass (HSU-0101152, MARIENFELD) with OptiMuS and covered with a coverslip to keep the tissue immersed in solution. The imaging was performed using a Plan-Apochromat 20× objective lens (NA 0.8 and WD 0.55 mm). The IMARIS software was subsequently used for the 3D image reconstruction.

#### LSFM

Fluorescence imaging of cleared tissues, including whole mouse brains, organs (kidney, heart and spleen), human tissue blocks and brain organoids, was performed using a light-sheet microscope (Light-Sheet 7, Zeiss) equipped with a 5× objective lens (NA 0.16) and a 20× objective lens (NA 1.0). The images were captured using two PCO Edge 4.2 scientific complementary metal–oxide–semiconductor (sCMOS) cameras. A thin light sheet was sequentially projected from each side of the sample, and the resulting images were merged for acquisition. Most samples, including α-SMA-stained brain (8 µm *z*-stack intervals), kidney (5 µm), heart (5 µm), tyrosine hydroxylase (TH)-stained mouse brain (8 µm), spleen (10 µm) and Tuj1 and HuC/D-stained intestine (4 µm), 4′,6-diamidino-2-phenylindole (DAPI) and GFAP-stained ChAT-Cre mouse brain (100 µm, 4 µm) and GFAP-stained rat brain (5 µm) were imaged using the 5× objective lens.

In addition, human brain blocks were imaged using the 5× objective lens, with Iba-1-stained blocks (8-µm *z*-stack intervals), MAP2-stained blocks (7 µm) and histone-H3-stained blocks (5 µm). For the brain organoids, the D50 sample was imaged with the 5× objective lens (5-µm *z*-stack intervals), whereas the D16 and D21 samples were imaged using the 20× objective lens with 1.5× zoom (3-µm intervals). The samples were immersed in OptiMuS within the sample chamber during imaging. Each dataset was acquired as a tiled sequence with a 10–20% overlap between adjacent tiles, which were then stitched into single images using the fuse tile function in Zen Blue software (Zeiss). IMARIS 6.0 (Bitplane AG) was used for 3D reconstruction and rendering of all *z*-stack images.

### Quantification of normalized fluorescence intensity

To quantify the intensity on the basis of the depth and the presence of urea, mouse brains were sliced into 3.5-mm sections, incubated in OptiMuS-prime clearing solution at 37 °C for 3 days to passively remove lipids and stained with lectin dye. The images were captured at 5-µm intervals using a confocal microscope with a Plan Apochromat 10× objective lens (NA 0.5 and WD 5.5 mm). The acquired images were analyzed in Fiji, where stained vascular signals from optical sections at 300-µm intervals were randomly selected, with ten signals per section. The average intensity value was measured, and all fluorescence signals were normalized to the 0-µm reference.

### Comparison of permeabilization and delipidation capacity of different detergents

The mouse half-brain (2 days), whole brain (3 days) and 7-mm-thick human brain tissue samples (4 days) were treated with the following detergents: 4% (w/v) SDS (1610302, BioRad), 10% (w/v) 3-((3-cholamidopropyl) dimethylammonio)-1-propanesulfonate (CHAPS; 1545, BioVision), 10% (w/v) SC, OptiMuS and OptMuS-prime. After incubation at 37 °C for the respective durations, the samples were washed with PBS and stained with 0.1% Coomassie blue (1610400, BioRad) at 37 °C for approximately 1 day. Following PBS washing, the samples were sliced to evaluate the dye penetration efficiency in the inner tissue, assessing both delipidation and permeabilization capabilities. We stained the samples with Coomassie blue and acquired red, green and blue (RGB) images using a commercial digital camera (NEX-3, Sony), which captures 24-bit color images (8 bits per channel). Because the blue regions appear darker than the surrounding regions in line scans, we directly inverted the images using ImageJ/Fiji software to enhance the visibility of the stained regions without separating the RGB images into individual channels. For quantitative analysis, we drew a line profile across the center of each sample and extracted the pixel intensity values along this line. These values, referred to as ‘pixel values’, represent the grayscale-equivalent luminance computed by ImageJ from the RGB image. The pixel values were then averaged to provide a reliable assessment of dye distribution throughout the samples.

### Other optical tissue clearing methods

The clearing time for whole mouse brain was determined according to the corresponding original report.

#### CLARITY

For hydrogel embedding, a monomer solution was prepared containing 4% PFA, 4% (w/w) acrylamide (A8887, Sigma), 0.05% (w/w) bisacrylamide (75821, USB Corporation) and 0.25% (w/w) VA-044 (2,2′-azobis(2-(2-imidazolin-2-yl)propane)dihydrochloride; 225-02111, Wako Pure Chemical). The whole mouse brains were incubated in the hydrogel solution under vacuum at 37 °C for 6 h. The samples were then immersed in 4% (w/v) SDS and cleared passively at 37 °C for 14 days. After extensive washing in PBS for 1 day, RI matching was performed using RIMS for 2 days at 37 °C. For 150-µm-thick Chat-Cre::tdTomato brain slices, the samples were incubated in CLARITY buffer for 2 min at 37 °C, briefly washed and immersed in RIMS for 2 min at RT.

#### CUBIC

Reagent 1 consisted of 25% (w/w) urea, 25% (w/w) *N*,*N*,*N*′,*N*′-tetrakis(2-hydroxypropyl)ethylenediamine (T0781, TCI, Japan) and 15% (w/w) Triton X-100 in double-distilled water. Reagent 2 contained 50% (w/w) sucrose (31365S0350, JUNSEI), 25% (w/w) urea and 10% (w/w) triethanolamine (T58300, Sigma) in double-distilled water. For whole mouse brain clearing, samples were incubated in reagent 1 for 7 days, washed for 1 day and then treated with reagent 2 for 2 days. For 150-µm-thick Chat-Cre::tdTomato brain slices, samples were incubated in reagent 1 for 2 min, briefly washed and then treated with reagent 2 for 2 min. All procedures were performed at 37 °C.

### Statistics and reproducibility

The statistical comparisons and graph creation were conducted using Prism 8.0 (GraphPad Software). The *P* values are provided in the figure legends, and the data are expressed as mean ± s.d., unless otherwise stated. All the experiments involving mice and humans were conducted with at least three independent replicates. This includes tissue size measurement, penetration tests (whole mouse brain and human brain block), signal-to-noise ratio (SNR) measurement and normalized fluorescence intensity measurement. For multiple group comparisons, an ordinary one-way analysis of variance, followed by Dunnett’s multiple comparison test, was performed. The statistical significance is indicated as follows; ***P < 0.001, **P < 0.01, *P < 0.05.

## Results

### Development of OptiMuS-prime

To address the limitations of SDS mentioned above and select the most suitable detergent for passive clearing, we screened several detergents commonly used in previous clearing methods for their delipidation capabilities during passive clearing, including SDS from CLARITY^13^, CHAPS from SHANNEL^18,31^ and SC from SCARF. Among the candidates, SC emerged as the most effective, achieving significant lipid removal with minimal tissue shrinkage, a key advantage for preserving tissue integrity during passive clearing. (Supplementary Fig. 1). SC is a bile salt detergent with a steroidal structure that has a hydrophobic convex side and a hydrophilic concave side and thus possesses facial amphiphilicity^32^. Compared with other detergents, SC possesses a higher CMC (14 mm) and a lower aggregation number (4–16), which allows it to form substantially smaller micelles than other detergents, including SDS^19,20^. Moreover, because it is a nondenaturing detergent, these characteristics allow SC to efficiently remove lipids while minimizing tissue damage and protein disruption.

However, we found that SC alone was insufficient for complete delipidation in deeper tissue regions, suggesting that additional components are needed to achieve optimal probe infiltration into the samples (Supplementary Fig. 2). Building on our previous development of OptiMuS^28^, which enables rapid tissue clearing without deformation due to urea’s hyperhydration properties—reducing light scattering and improving tissue penetration—we hypothesized that combining SC with urea would synergistically enhance tissue clearing by leveraging SC’s efficient delipidation alongside urea’s ability to disrupt hydrogen bonds and induce hyperhydration.

**Fig. 1:**
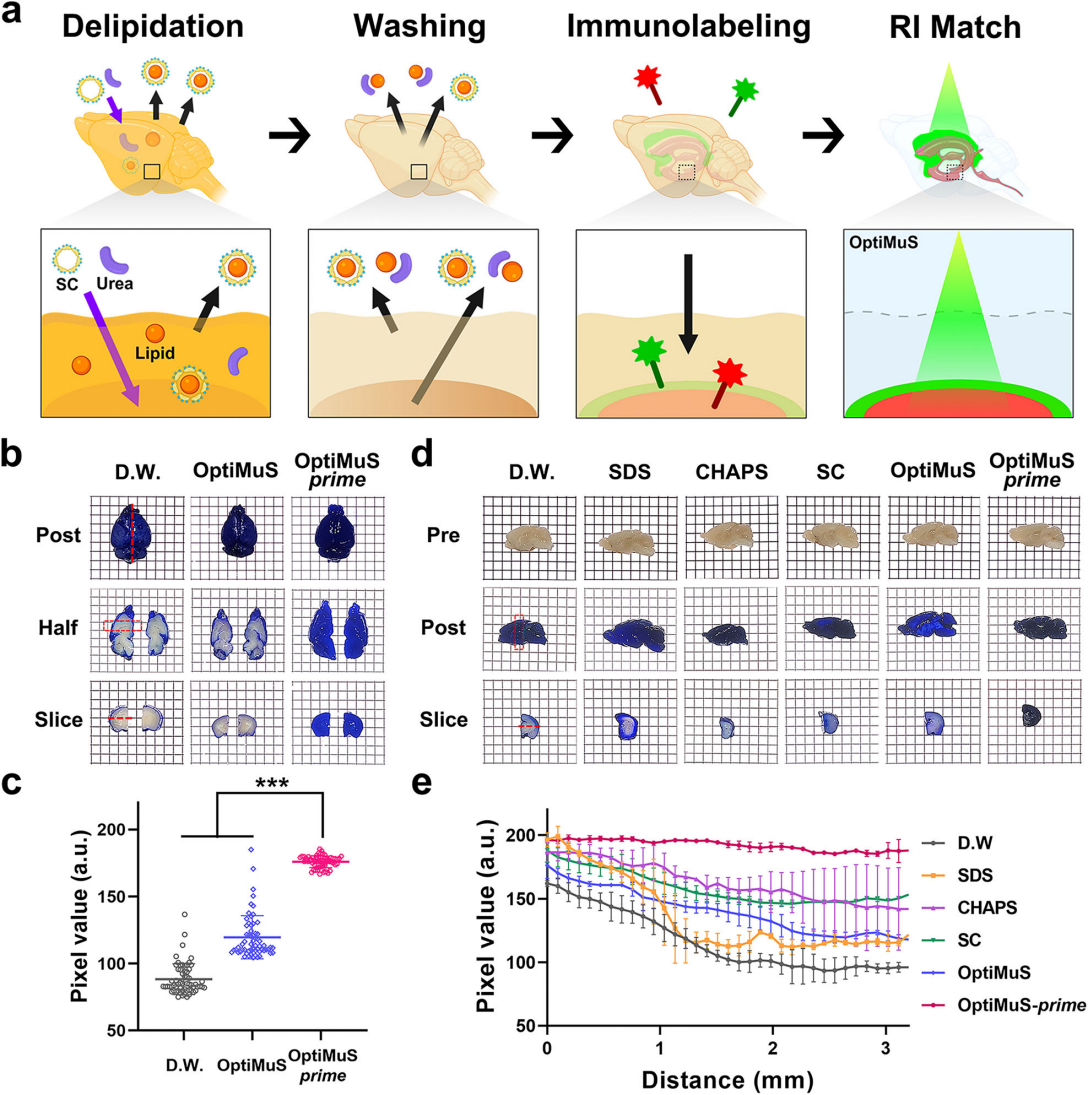
OptiMuS-prime demonstrates enhanced delipidation efficiency compared with conventional detergents. **a** A schematic diagram of the OptiMuS-prime process, including delipidation, washing, immunolabeling and RI matching. **b** The brightfield images of the whole mouse brain stained with Coomassie blue, as part of a penetration test, with a comparison of OptiMuS and OptiMuS*-*prime. The dotted red line marks the section along the line. Grid size: 2.5 mm × 2.5 mm. D.W., distilled water. **c** The intensity profile along the distance created by drawing a red line on the slice image in **b**. **d** The brightfield images of half mouse brains stained with Coomassie blue after delipidation with each detergent, performed to assess the delipidation efficiency. Pre (top), post (middle) and slice (bottom). The dotted red line indicates the section along the line. Grid size: 2.5 mm × 2.5 mm. **e** The intensity plot along the distance created by drawing a red line on the slice image in **e**. The data are shown as the mean ± s.d. (*n* = 3). ***P < 0.001, **P < 0.01, *P < 0.05.

Indeed, we found that the addition of urea significantly enhanced both lipid removal and probe penetration compared with SC alone (Supplementary Fig. 2), underscoring urea’s critical role in enabling deeper tissue penetration and effective clearing. Subsequent extensive optimization of the solution composition led to the development of OptiMuS-prime, a new clearing method that incorporates SC and urea for a superior passive tissue clearing, immunostaining and preservation of tissue integrity (Fig. 1a).

To evaluate the delipidation effectiveness of OptiMuS-prime, we conducted a penetration test by staining the whole mouse brain with Coomassie blue for 1 day after clearing with OptiMuS or OptiMuS-prime for 3 days. The results showed that OptiMuS-prime enabled the complete penetration of the Coomassie blue dye throughout the entire brain, whereas with OptiMuS, which lacks delipidating property, only the surface areas were stained (Fig. 1b, c). We next compared the delipidating capabilities of various detergents with OptiMuS-prime using mouse half-brains. The brains were incubated with the respective delipidating reagents for 2 days and stained with Coomassie blue for 1 day (Fig. 1d). We found that OptiMuS-prime demonstrated markedly higher staining intensity in the deeper regions of the brain tissue compared with the other detergents, underscoring its enhanced delipidation efficiency (Fig. 1e). In addition, after delipidation, we quantitatively measured the signal-to-noise ratio over the imaging depth and confirmed that OptiMuS-prime preserved signals from endogenous fluorescent proteins across the entire imaging depth, with negligible signal loss in the 3.5-mm-thick Thy1-EYFP mouse brain slices (Supplementary Fig. 3).

To further validate the clearing and size preservation capabilities of OptiMuS-prime, we directly compared it with other widely used aqueous-based clearing methods, including CUBIC^33^ and CLARITY, using the whole mouse brain and mouse brain sections. First, we cleared whole mouse brains with OptiMuS-prime, CUBIC and CLARITY, then quantitatively compared optical transparency and tissue size among the methods. OptiMuS-prime achieved higher light transmittance than CLARITY and was comparable to CUBIC (Supplementary Fig. 4). Importantly, OptiMuS-prime demonstrated superior size retention compared with both CUBIC and CLARITY, which caused noticeable tissue expansion (Supplementary Fig. 4). Although both OptiMuS-prime and CUBIC achieve similar measured transmittance values, CUBIC enhances transparency through a combination of chemical clearing and tissue expansion, demonstrating higher optical clearing efficiency per unit thickness due to the reduction in the attenuation coefficient associated with the expansion. By contrast, OptiMuS-prime achieves comparable transmittance without inducing tissue expansion, reflecting a robust chemical clearing capacity. This provides a practical advantage for applications where the preservation of original tissue dimensions and precise morphological analysis are essential.

At the cellular level, we tested 150-μm-thick coronal sections from the ChAT-Cre::tdTomato transgenic mice to assess the tissue distortion and preservation of internal architecture. We found that OptiMuS-prime-treated sections maintained their original dimensions and showed well-preserved neuronal morphology, with a clear visualization of cholinergic cell bodies. By contrast, sections cleared with CUBIC or CLARITY exhibited substantial tissue expansion and structural deformation (Supplementary Fig. 5).

Together, these results highlight the superior clearing performance of OptiMuS-prime across tissue scales from individual cells to whole organs, enabling the high transparency and structural integrity.

### OptiMuS-prime enables successful staining with various dyes and antibodies

**Fig. 2:**
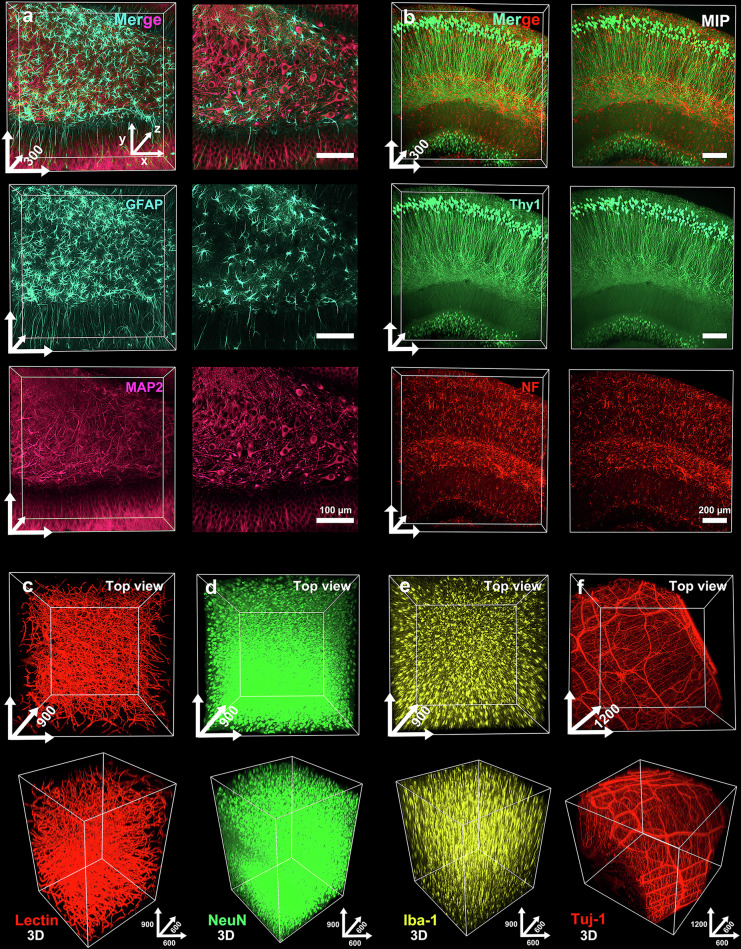
OptiMuS-prime demonstrates broad compatibility with a wide range of antibodies and fluorescent dyes, enabling consistent and reliable immunolabeling. **a** The 3D reconstruction and optical section images of 300-µm-thick mouse brain samples that were cleared with OptiMuS-prime and stained with anti-GFAP antibody and anti-MAP2 antibody. Merge (top), anti-GFAP (middle) and anti-MAP2 (bottom). Left: optical section at 56-μm depth. Scale bar in optical sections, 100 µm. **b** The 3D reconstruction and maximum intensity *z*-projection (MIP) images of 300-µm-thick Thy1-EYFP transgenic mouse brain samples stained with anti-neurofilament (NF) antibody. Merge (top), Thy1 (middle) and anti-NF antibody (bottom). Scale bar in MIPs, 200 µm. **c**–**e** The 3D reconstruction images of 1-mm-thick mouse brain section stained with fluorophore-conjugated *Lycopersicon esculentum* (tomato) lectin, a blood vessel marker (**c**), anti-NeuN, a neuronal marker (**d**) and anti-iba1, a microglia marker (**e**). **f** The 3D reconstruction images of the 1,200-µm-thick mouse intestine sample stained with anti-Tuj1 (neuron-specific class III beta-tubulin) antibody. The 3D axis is expressed in µm. The scale bars for the *x* and *y* axes in 3D coordinates of figures represent 100 µm (**a**) and 200 µm for **b**–**f** whereas the *z* axis indicates full tissue depth (μm).

We subsequently tested whether OptiMuS-prime enables immunolabeling with dyes or antibodies. We sectioned the mouse brains and performed antibody staining, followed by imaging with confocal microscopy. The 300-µm-thick hippocampal brain sections from Thy1-EYFP transgenic mice were passively cleared with OptiMuS-prime for 3 h and stained with anti-GFAP antibody (an astrocyte marker) and either anti-MAP2 (a neuronal marker) (Fig. 2a) or anti-neurofilaments antibody (Fig. 2b). We found that the fine details of glial and neuronal processes were clearly observed throughout the entire imaging depth at the single-cell resolution without compromising the image quality.

Next, OptiMuS-prime was applied to clear 1-mm-thick brain tissue sections, which were then stained with lectin dye and either anti-NeuN, anti-Iba-1 or anti-Tuj1 antibody. The fine vascular and neural structures, including cell bodies and neurites, were well observed throughout the entire depth of the brain (Fig. 2c–e). OptiMuS-prime was also successfully applied to clear and immunostain enteric nerve processes in the mouse intestine using the anti-Tuj1 antibody (Fig. 2f).

### OptiMuS-prime enables 3D imaging of arteriolar structures in various mouse organs

Next, we applied OptiMuS-prime to clear and immunostain whole mouse organs. After delipidation with OptiMuS-prime, we stained the organs with the anti-α-SMA antibody to visualize vascular structures, followed by whole-organ 3D volume imaging using light-sheet fluorescence microscopy (LSFM).

**Fig. 3:**
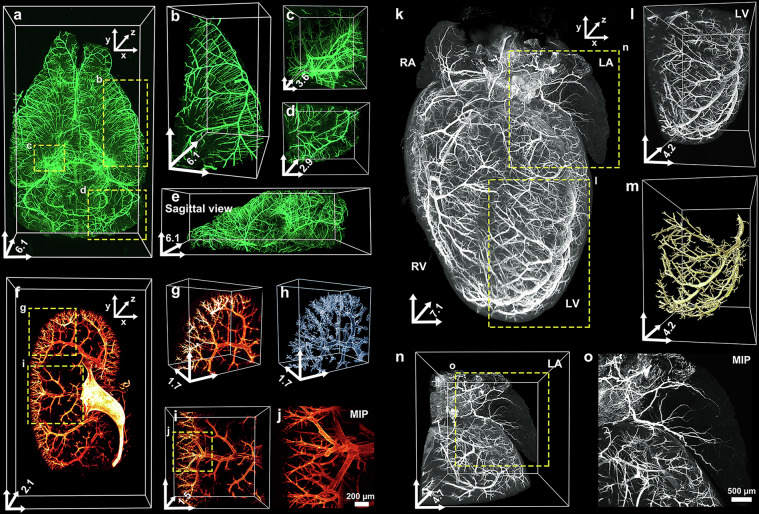
OptiMuS-prime enables 3D visualization of arteriolar structures across various mouse organs. **a** The 3D view image of alpha smooth muscle (α-SMA)-stained mouse whole brain processed by OptiMuS-prime. **b**–**d** The detailed arteriolar structures in 3D images of brain regions including the cortex (**b**), hippocampus (**c**) and cerebellum (**d**) corresponding to the regions highlighted by rectangles in **a**. **e** The sagittal view of the reconstructed brain shown in **a**. **f** A 3D reconstruction of the α-SMA-immunostained vasculature in a mouse hemikidney. **g**, **h** The magnified views of the 3D image (**g**) and 3D-rendered reconstruction image (**h**) of glomerular tufts, corresponding to the region enclosed by the rectangle (labeled **g**) in **f**. **i** The magnified views of the 3D kidney vasculature, corresponding to the region enclosed within the rectangle (labeled **i**) in **f**. **j** The maximum intensity *z*-projection (MIP) image of the rectangle in **i**. Scale bar, 200 µm. **k** The 3D-view image of the α-SMA-stained mouse heart. Scale bar, 1 mm. **l** A detailed 3D image of left ventricle (LV) vasculature corresponding to the region highlighted in **k**. **m** A 3D-rendered reconstruction image of the LV vasculature in **l**. **n** A detailed 3D image of the left atrium (LA) vasculature corresponding to the region highlighted in **k**. **o** The MIP image of the LA in **n**. Scale bar, 500 µm. The scale bars for the *x* and *y* axes in 3D coordinates of figures represent 2 mm in **a**, **b** and **e** 1 mm in **f**–**i** and **k**–**n** and 500 µm in **c** and **d** whereas the *z* axis indicates the full tissue depth (mm). RA, right atrium; RV, right ventricle.

We found that the major α-SMA-positive vascular structures, including arterioles, in the whole mouse brain were well resolved (Fig. 3a). The enlarged images revealed α-SMA-positive arteriolar networks throughout the entire 3D depth of the cortex, hippocampus and cerebellum (Fig. 3b–d), and the distribution of blood vessels along the *z* axis in sagittal view were clearly visualized and easily traced in three dimensions (Fig. 3e).

We also observed that arteriolar structures in the whole mouse kidney and heart were clearly visualized with the anti-α-SMA antibody staining after OptiMuS-prime clearing (Fig. 3f, k). The enlarged and 3D-rendered images revealed the detailed arteriolar distribution in the kidney (Fig. 3f–j). Furthermore, we confirmed that the arteriolar structures of the left atrium and left ventricle in the heart can also be reconstructed and rendered in 3D (Fig. 3k–o). Moreover, the larger α-SMA-positive vascular structures in the whole lung were also clearly visualized (Supplementary Fig. 6).

### OptiMuS-prime enables 3D visualization of neuronal network at single-cell level resolution

**Fig. 4:**
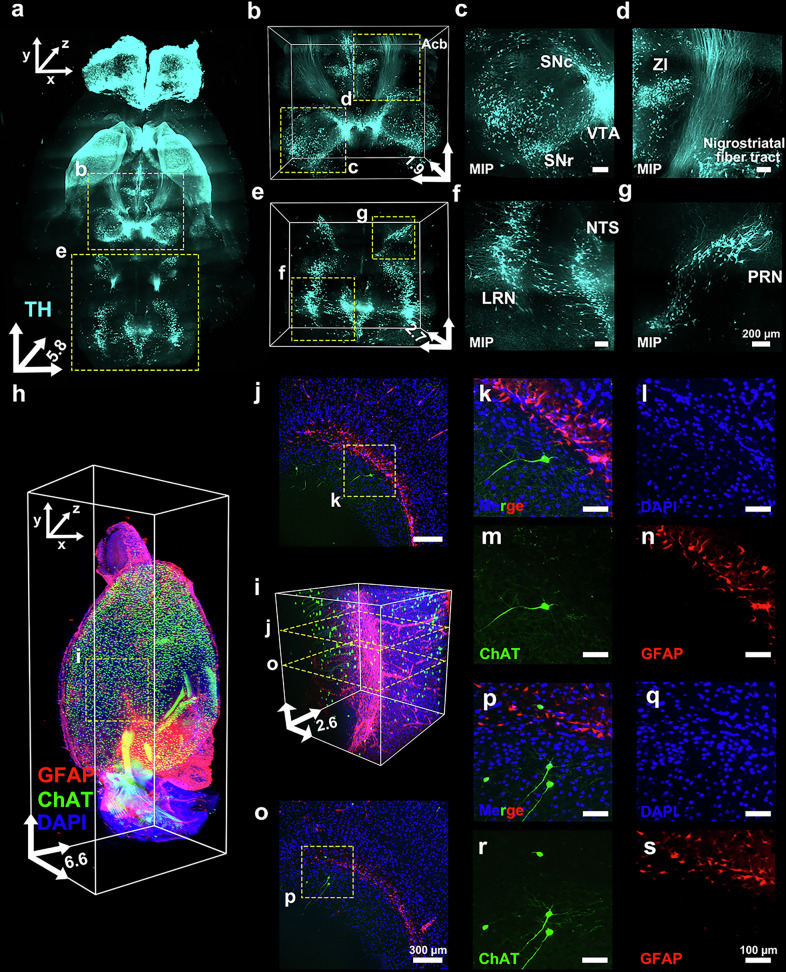
OptiMuS-prime enables comprehensive 3D visualization through efficient labeling of neuronal markers in various mouse tissues. **a** A 3D-view image of anti-TH stained mouse whole brain processed by OptiMuS-prime. Scale bar, 2 mm. **b** The magnified views of the 3D reconstruction image corresponding the region highlighted in **a**. **c** The magnified maximum intensity *z*-projection (MIP) images of TH-positive areas in the VTA, substantia nigra pars reticulata (SNr) and SNc, corresponding to the region highlighted in **b**. Scale bar, 200 µm. **d** A magnified MIP image of TH-positive nigrostriatal fiber tracts and zona incerta (ZI), corresponding to the regions highlighted in **b**. Scale bar, 200 µm. **e** The magnified views of the 3D reconstruction image corresponding the region highlighted in **a**. **f** The magnified MIP images of TH-positive areas in the LRN and NTS, corresponding to the region highlighted in **e**. Scale bar, 200 µm. **g** The magnified MIP images of TH-positive areas in the PRN, corresponding to the region highlighted in **e**. Scale bar, 200 µm. **h** A 3D view of a half mouse brain from a ChAT-Cre::tdTomato transgenic mouse, stained with anti-GFAP and DAPI. The whole mouse brain was cleared using OptiMuS-prime (see Supplementary Fig. 7h for the cleared whole brain), immunostained and halved, and one hemisphere was imaged. **i**, The 3D reconstruction image of the hippcocampal region corresponding to the area highlighted in **h**. Scale bar, 300 µm. **j**, **o** The representative optical section images at 1,660-µm depth (**j**) and 1,436-µm depth (**o**) from the 3D reconstruction in **i**. **k**–**n**,**p**–**s** The magnified images of regions highlighted in (**j**): Merge (**k**), DAPI (**l**), ChAT (**m**), and GFAP (**n**); and of the regions highlighted in (**o**): Merge (**p**), DAPI (**q**), ChAT (**r**), and GFAP (**s**). Scale bar, 100 µm. The scale bars for the *x* and *y* axes in the 3D coordinates of figures represent 2 mm in **a** and **h**, 1 mm in **b** and **e** and 500 µm in **i**, whereas the *z* axis indicates full tissue depth (mm).

Next, the whole mouse brain was delipidated with OptiMuS-prime and stained with anti-TH antibody to trace the dopaminergic tracts using LSFM. The dopaminergic pathways were clearly identifiable in 3D whole-brain imaging, with intense anti-TH staining in the substantia nigra pars compacta (SNc) along with projections to the caudate–putamen in the basal ganglia^34^ (Fig. 4a). The positive TH staining also highlighted the mesolimbic pathway, extending from the ventral tegmental area (VTA) to the nucleus accumbens (Acb) (Fig. 4b–d). The TH expression was also robust in regions, with notable presence in the nucleus of the solitary tract (NTS), lateral reticular nucleus (LRN) and pontine reticular formation (PRN) (Fig. 4e–g).

In addition, the TH staining clearly visualized the 3D neural architecture of sympathetic neurons in the spleen, successfully capturing the intricate, panicle-shaped of the autonomic nervous system in 3D (Supplementary Fig. 7a–d). OptiMuS-prime further enabled us to visualize the enteric neural network structure within the intestinal wall by staining nerve fibers and cell somata using anti-Tuj1 and anti-HuC/D (marker for RNA-binding proteins) antibodies (Supplementary Fig. 7e–g).

We also applied OptiMuS-prime to the Chat-Cre::tdTomato mice brain to achieve tissue clearing while preserving fluorescent protein signals through delipidation. We then performed coimmunostaining with DAPI (a nuclear marker) and an anti-GFAP antibody. Following tissue clearing, the whole brain was divided into left and right hemispheres, and one hemisphere was imaged using LSFM, enabling the successful 3D visualization of the entire cleared half-brain as well as the hippocampal region in detail (Fig. 4h, i and Supplementary Fig. 7h). The coimmunostaining with DAPI and an anti-GFAP antibody enabled the clear identification of tdTomato-positive cholinergic neurons and GFAP-positive astrocytes at the single-cell resolution (Fig. 4j–s). This allowed the precise localization of individual cell structures and nuclear positions in 3D space. These results demonstrate that the OptiMuS-prime enables the high-resolution, simultaneous 3D volumetric imaging of neuronal and glial populations in the whole mouse brain.

Furthermore, we extended the application of OptiMuS-prime to whole rat brains (approximately 2.5 cm long, 1.5 cm wide and 1.1 cm high) to assess its scalability for larger tissue samples. After delipidation with OptiMuS-prime for 7 days, we found that the large rat brain was successfully cleared (Supplementary Fig. 8a). We then immunostained it with an anti-GFAP antibody to label astrocytes. The cleared brain was coronally sectioned, and each section was subsequently imaged using LSFM. High-resolution imaging of the hippocampal region revealed well-preserved astrocytic morphology, demonstrating that OptiMuS-prime is compatible with larger brain tissues while maintaining cellular-level resolution (Supplementary Fig. 8b–e).

### OptiMuS-prime is successfully applicable for clearing and immunostaining of densely packed post-mortem human tissues

Post-mortem human brain samples are challenging to clear and stain owing to high myelination and dense, opaque molecules, often taking months to process even small pieces^15,18,35^. Moreover, long-term formalin fixation masks antigens and forms cross-links, hindering staining probe penetration^36,37^.

We conducted a Coomassie blue penetration assay to evaluate the delipidation capability of OptiMuS-prime on post-mortem human brain samples (Supplementary Fig. 9a) and found, similar to the results in mouse brain samples, that OptiMuS-prime exhibited significantly higher staining intensity throughout the internal regions of the post-mortem human brain block compared with the other detergents (Supplementary Fig. 9b).

**Fig. 5:**
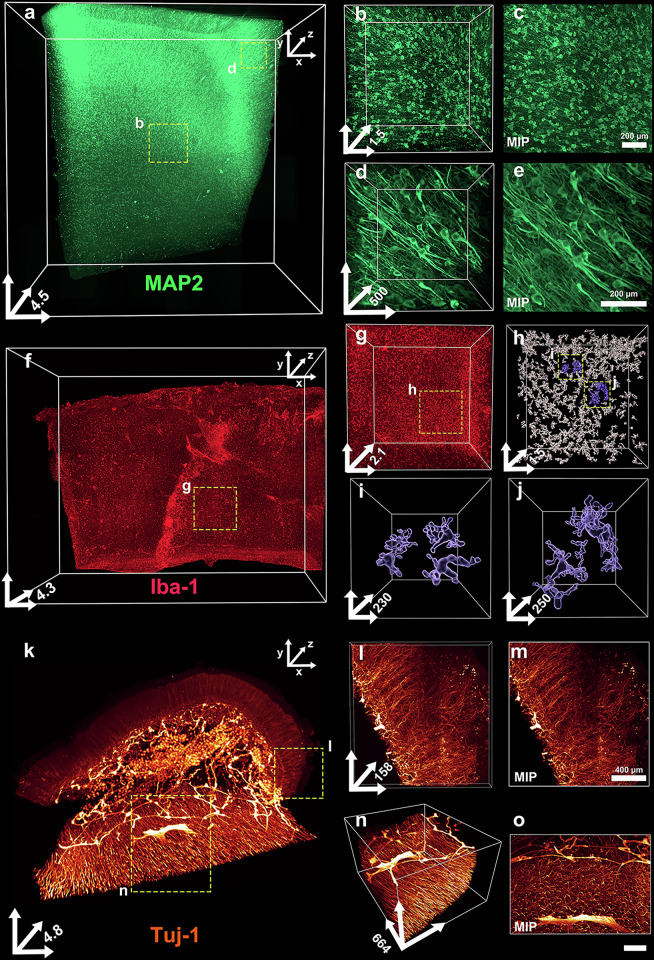
OptiMuS-prime demonstrates high efficacy in tissue clearing and immunostaining of densely packed post-mortem human tissues. **a** A 3D reconstruction image of a MAP2-stained post-mortem human brain block processed using OptiMuS-prime. **b**, **d** A magnified 3D image corresponding to the regions highlighted by rectangles (**b**) and (**d**) in **a**. **c**, **e** The maximum intensity z-projection (MIP) image of b is shown in **c**, and that of **d** is shown in **e**. Scale bar, 200 µm. **f** A 3D reconstruction image of an Iba-1-stained post-mortem human brain block processed using OptiMuS-prime. **g** A magnified 3D image of microglia corresponding to the region highlighted in **f**. **h** The detailed 3D-rendered reconstruction images corresponding to the region highlighted in **g**. **i**, **j** Detailed 3D-rendered reconstruction images of microglia at single-cell resolution, corresponding to the regions highlighted in **h**, with **i** and **j** showing two separate highlighted regions. **k** A 3D reconstruction image of Tuj1-stained post-mortem human colon processed using OptiMuS-prime. **l**–**o** The magnified 3D images from the regions highlighted in **k** are shown in **l** and **n**, and the corresponding MIP images are shown in **m** and **o**. Scale bar, 200 µm. The scale bars for the *x* and *y* axes in 3D coordinates of figures represent 1 mm in **a**, **f** and **k**, 500 µm in **g**, 400 µm in **l** and **n**, 200 µm in **b**, **d** and **h** and 50 µm in **i** and **j**, whereas the *z* axis indicates full tissue depth in mm in **a**, **b**, **f**, **g**, **h** and **k** and in µm in **d**, **i**, **j**, **l** and **n**.

Next, 3–5-mm-thick post-mortem human brain cortex samples were first decolorized, passively cleared using OptiMuS-prime for 4 days, immunostained with anti-MAP2 and anti-Iba-1 antibodies and then imaged using LSFM (Fig. 5a, f). OptiMuS-prime enabled the visualization of neural network microglia at the single-cell resolution throughout the entire tissue depth (Fig. 5b–e, g–j). For high-resolution confocal imaging, 1-mm-thick human brain samples were cleared using OptiMuS-prime, immunostained with anti-MAP2, anti-GFAP antibodies and anti-Histone H3 antibody, a marker for cell proliferation and nuclear organization (Supplementary Fig. 10). These results indicate that OptiMuS-prime enabled robust immunostaining and improved the performance of challenging antibodies, such as those targeting subcellular structures and nuclear proteins. In addition, post-mortem human colon tissue cleared with OptiMuS-prime enabled the 3D visualization of Tuj1-positive nerve fibers throughout the specimen (Fig. 5k–o).

### OptiMuS-prime efficiently clears, immunostains and enables 3D imaging of human brain organoids at various developmental stages

Human brain organoids develop from two-dimensional induced pluripotent stem cells cultures through 3D embryoid bodies, which differentiate into clusters of neural progenitor cells known as neural rosettes^38,39^. The neural progenitor cells located at the rosette base undergo mitosis and differentiation to develop into mature neurons, forming neural rosettes by around D16 and neuronal layers on the surface of the organoid by D50. The 3D visualization of brain organoids is essential for understanding neuronal differentiation, migration and cortical layering. As the organoids increase in size, the increasing density of their internal structures limits optical accessibility, thus necessitating advanced tissue clearing techniques for intact 3D imaging without sectioning^40–42^. Due to the high density of internal structures, however, previous attempts to clear organoids often rely on harsh organic reagents that can compromise the integrity of organoids.

**Fig. 6:**
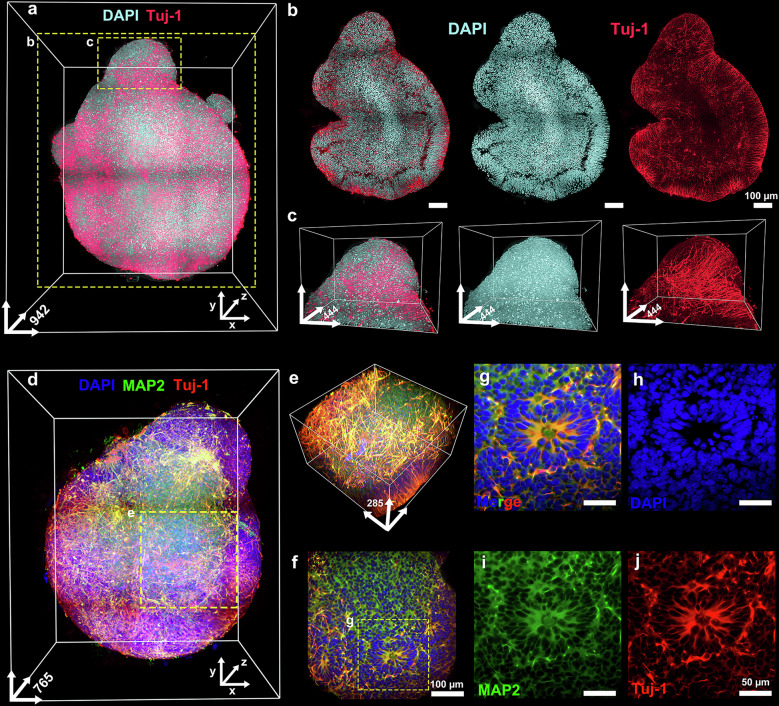
OptiMuS-prime exhibits high efficacy in tissue clearing, immunostaining and enabling 3D imaging of human brain organoids. **a** A 3D reconstruction image of D16 human brain organoids stained with DAPI and Tuj1 after processing with OptiMuS-prime. **b** The optical section images of human brain organoids at 573-μm depth, corresponding to the region highlighted by the rectangle (labeled **b**) in **a**. Merge (left), DAPI (middle) and Tuj1 (right). Scale bar, 100 µm. **c** The detailed 3D images of human organoids corresponding to the region highlighted by rectangle (labeled **c**) in **a**. Merge (left), DAPI (middle) and Tuj1 (right). **d** A 3D reconstruction image of D16 human brain organoids costained with DAPI, MAP2 and Tuj1 after processing with OptiMuS-prime. **e** A detailed 3D reconstruction image corresponding to the region highlighted by the rectangle in **d**. **f** A representative optical section image at 249-μm depth from **e**. Scale bar, 100 µm. **g**–**j** The magnified images of progenitor cells corresponding to the region highlighted by the rectangle in **f**: merge (**g**), DAPI (**h**), MAP2 (**i**) and Tuj1 (**j**). Scale bar, 50 µm. The scale bars for the *x* and *y* axes in 3D coordinates of all figures represent 100 µm unless indicated otherwise, whereas the *z* axis indicates full tissue depth (µm).

OptiMuS-prime clearing of D16 human brain organoids, followed by immunostaining with anti-Tuj1 and DAPI labeling, enabled the complete 3D visualization of organoids (Fig. 6a). High-magnification images and optical section images clearly revealed the neuronal arrangement and cellular architecture within the organoid in detail (Fig. 6b, c).

Tuj1 imaging of D21 brain organoids also revealed early neural development patterns, with DAPI labeling demonstrating nuclear distribution as part of the overall structural organization (Supplementary Fig. 11a). We visualized the 3D structures of human brain organoids by costaining with DAPI, Tuj1 and MAP2 (Fig. 6d). High-magnification images revealed well-defined clusters of neural progenitor cells and early neuronal structures, confirming the distinct cellular patterns within the organoids (Fig. 6e–j). We further successfully applied OptiMuS-prime to visualize the 3D distribution patterns of protein and cell nuclei in D50 human brain organoids using N-hydroxysuccinimide (NHS)-ester dye and SYTO-X staining (Supplementary Fig. 11b).

## Discussion

In this Article, we aimed to address the limitations of passive clearing methods based on SDS-mediated delipidation and provide a more reliable and accessible alternative for passive tissue clearing. OptiMuS-prime combines SC with urea to achieve an optimal balance between delipidation of thick samples by enhancing penetration and preserving protein epitopes and tissue integrity.

Because SC performs superiorly in delipidation compared with SDS^17,27^, we initially expected that SC would achieve efficient transparency in the passive clearing process. Although it worked well, we found that SC alone was not sufficient for the effective clearing of deep tissues (Supplementary Fig. 2). We found that the addition of urea is critical, as it synergistically enhances SC’s delipidation capability by disrupting hydrogen bonds and inducing hyperhydration. This significantly improves the antibody penetration and clearing performance. This new protocol enables the efficient clearing, immunostaining and 3D imaging of a variety of biological samples.

OptiMuS-prime addresses several key challenges of existing passive tissue clearing methods. Hydrophobic clearing techniques, such as those using organic solvents, achieve high transparency but often cause substantial tissue shrinkage and fluorescence loss due to dehydration^43–45^. By contrast, hydrophilic clearing methods, despite preserving fluorescence and being more biosafe, generally result in lower optical transparency^3,46^. As a water-based solution, OptiMuS-prime overcomes these limitations by combining SC’s effective delipidation with urea’s superior penetration properties. This synergy enhances lipid removal, improves antibody penetration into deeper tissue regions and ensures the preservation of both tissue structure and epitopes.

The versatility of OptiMuS-prime is demonstrated by its successful application across various tissue types, enabling immunolabeling with antibodies such as anti-α-SMA, anti-TH, anti-GFAP and anti-Tuj1, as well as 3D visualization in mouse brain, heart, kidney and lung tissues. Combined with LSFM, OptiMuS-prime enabled detailed visualization of α-SMA-positive arteriolar structures, neuronal networks and other cellular components at single-cell resolution. Indeed, the clear visualization of dopaminergic pathways in the brain and the detailed 3D reconstruction of sympathetic neurons in the spleen underscore the potential of OptiMuS-prime in neurobiological research. In addition, OptiMuS-prime enables the efficient clearing, immunostaining and imaging of densely packed post-mortem human tissues and whole organoids, simplifying the 3D visualization of their structural organization and cellular distribution. This capability has the potential to drive new discoveries in areas such as disease modeling, drug response studies and tissue development^47^. By integrating OptiMuS-prime with organoid imaging, we expect to achieve deeper insights into disease mechanisms, enabling a more detailed visualization of cellular processes and pathological changes.

Despite its advantages, OptiMuS-prime has several limitations that should be considered. First, as a passive clearing method that relies exclusively on diffusion-based processes, compared with active clearing methods that utilize external forces such as electrical fields, OptiMuS-prime is intrinsically slower, requiring extended processing time ranging from several days to weeks. For larger samples, it would take more processing time and may require additional exchanges of clearing solution to efficiently wash off removed lipids and ensure better diffusion throughout the tissue. It does, however, substantially reduce the risk of structural damage, thereby preserving tissue architecture and molecular information with higher fidelity. For researchers prioritizing processing speed over structural integrity, active clearing methods such as CLARITY or DISCO-series protocols may be more suitable alternatives. Second, although the protocol is broadly compatible with various tissues and organs, its clearing performance can vary depending on the tissue type and lipid content. Furthermore, its performance may vary depending on the specimen age and physiological conditions, mainly due to differences in tissue composition, lipid content and extracellular matrix properties. Achieving uniform clearing and immunolabeling under these conditions necessitates tissue-specific optimization. Therefore, further careful optimization and standardization are needed to ensure consistent results across different biological samples. Third, OptiMuS-prime uses urea for its hyperhydration effect to facilitate the tissue penetration of SC. However, urea, especially at high concentrations, may damage the structural integrity of intact tissues, potentially leading to abnormal swelling or deformation of hydrophilic molecules and components^48–50^. These effects should be carefully considered when interpreting experimental results. Finally, the application of OptiMuS-prime to RNA detection remains unexplored. Integrating tissue clearing with RNA detection has advanced spatial transcriptomics, allowing the visualization of both anatomical structures and gene expression patterns within intact tissues. RNA molecules, however, require specialized fixation and preservation conditions owing to their fragility and susceptibility to degradation during processing^51,52^. Because most current tissue clearing methods, including OptiMuS-prime, were originally developed for protein detection, adapting them to RNA detection requires substantial modifications to clearing protocols, including specialized fixation and handling procedures. For example, CLARITY incorporated 1-ethyl-3-(3-dimethyl-aminopropyl)carbodiimide (EDC) fixation to retain RNA during hydrogel-based clearing^53^. In another approach, Tris-buffer-mediated retention of in situ hybridization chain reaction signal in cleared organs (TRISCO) improved RNA retention and tissue transparency by replacing saline-sodium citrate (SSC) buffers with high-concentration Tris-HCl buffer^54^. DNA probes rather than RNA probes for better penetration were used for faster fluorescence in situ hybridization (FISH) protocols^53,54^. Similar adaptations would probably be necessary for OptiMuS-prime, potentially including enhanced fixation methods, optimized probe selection and shortened processing times. Despite these challenges, extending OptiMuS-prime for RNA-based applications such as spatial transcriptomics represents a promising future direction that could support multimodal tissue analysis while maintaining the method’s inherent simplicity and accessibility.

OptiMuS-prime provides effective 3D volume imaging at both cellular and subcellular levels while maintaining tissue architecture integrity. Its accessible protocol requires no specialized equipment or advanced technical expertise, making it adaptable for laboratories with diverse resources. We anticipate that OptiMuS-prime will readily complement standard immunocytochemistry and immunohistochemistry workflows, offering improved visualization with reduced light scattering. Future investigations might explore its application in combining protein detection with RNA visualization techniques, potentially expanding its utility for mapping molecular distributions in intact specimens. With continued refinement, OptiMuS-prime could serve as a valuable tool for researchers across various biological disciplines seeking to examine spatial relationships within complex tissues.

## Supplementary information

Supplementary Information

## Data Availability

The data that support the findings of this study are available from the corresponding author upon reasonable request.
