# Supplementary material for: A novel protein-preserving passive tissue clearing approach using sodium cholate and urea for whole-organ imaging
Source: Exp Mol Med. 2025 Oct 1;57(10):2292–304. doi: 10.1038/s12276-025-01550-w (PMC12586516; doi:10.1038/s12276-025-01550-w)
Supplement: Supplementary file 1 — Supplementary Information [file 12276_2025_1550_MOESM1_ESM.pdf]

SUPPPLEMENTARY INFORMATION

A Novel Protein-preserving Passive Tissue Clearing Approach Using Sodium Cholate and Urea for Whole-organ Imaging

Kitae Kim[1], Kwangbae Lee[1], Taehoon Kang[1], Jungmihn Lee[1], Wonseok Lee[2,3,4], Ji Yeoun Lee[2,5,6] and Sunghoe Chang[1,6]*

[1]Department of Physiology and Biomedical Sciences Seoul National University College of Medicine, [2]Department of Anatomy and Cell Biology Seoul National University College of Medicine, [3]Department of Transitional Medicine Seoul National University College of Medicine, [4]Department of Neurosurgery Seoul National University College of Medicine, [5]Division of Pediatric Neurosurgery, Seoul National University Children's Hospital, [6]Neuroscience Research Institute, Seoul National University College of Medicine, 103 Daehak-ro, Jongno-gu, 03080 Seoul, South Korea

* Corresponding author e-mail address: sunghoe@snu.ac.kr

Supplementary Methods

Quantitative measurement of linear expansion

Bright-field images were captured using a commercial camera (NEX-3, Sony, Japan) to measure changes in sample size during the clearing process. For the 1 mm-thick mouse brain slices, measurements were performed at three stages: pre-treatment, delipidation, and post-treatment (RI matching). For the 150 μm-thick mouse brain slices and whole mouse brains, measurements were taken only before and after the clearing step. The area of the brain samples was determined from top-view photographs using the 'Polygon Selection' function in the ImageJ/Fiji program (National Institutes of Health, USA). Linear expansion was calculated by taking the square root of the change in area, with the pre-treatment measurement used as the reference.

Quantitative Measurement of area size change

To assess the structural integrity of neuronal somata, brain slices (150-μm thickness) from Chat-Cre::tdTomato mice were imaged both before and after tissue clearing at identical positions. Images were processed in Fiji, where identical regions of interest (ROIs) encompassing neuronal cell bodies were cropped and aligned via rigid registration. The area of each neuronal soma was measured by manually tracing its outline using the 'Polygon Selection' tool in ImageJ/Fiji. Neuronal somata area size changes were quantified by calculating the percentage difference before and after clearing.

Measurement of SNR

Thy1-EYFP transgenic mouse brains were sliced into 3.5 mm thick sections, and EYFP fluorescence images were acquired at 5 μm intervals after delipidation. Imaging was performed using a confocal microscope with a Plan Apochromat 10× objective lens (NA 0.5, WD 5.5 mm). The acquired images were then transferred to Fiji for further analysis. To assess the signal-to-noise ratio (SNR), five dendrites were manually selected from single optical section images obtained at 300 μm intervals from the tissue surface to a depth of 3,300 μm. Dendrites were identified based on clearly visible EYFP fluorescence signals as determined by visual inspection without applying post-acquisition thresholding. In each image, a separate region of interest (ROI) of equivalent size to the dendrite ROIs was selected in an adjacent area containing only background signal. We measured the mean pixel intensity in the EYFP-

positive dendritic regions and the mean pixel intensity in the background ROIs. Noise was estimated as the standard deviation of the background pixel intensities. SNR was calculated by subtracting the background signal from the dendritic EYFP signal and dividing the result by the background noise.

Quantitative measurement of transmittance

For transmittance measurements, the optical transparency of whole mouse brains was assessed using a spectrophotometer (UV mini-1240, Shimadzu, Japan). Cleared brains were placed in a cuvette and positioned so that the light beam passed through the central region of the brain. Transmittance was measured at 20 nm intervals across the 400–800 nm wavelength range. A blank value was obtained using only the clearing reagent without tissue, and the transmittance of each sample was normalized to this blank.

Imaging

1. Confocal fluorescence microscopy

Fluorescence imaging of 150 μm-thick ChAT-Cre::tdTomato brain slices and the D50 brain organoid section was performed by placing the sample on a slide glass and imaging it with a spinning disk confocal microscope (ECLIPSE Ti-E, Nikon). An oil immersion Plan Apochromat 40× objective lens (N.A. = 1.30) and a Neo sCMOS camera (Andor Technology) were used, with image acquisition controlled by NIS-Elements software.

2. Light-sheet fluorescence microscopy

Fluorescence imaging of a cleared, α-SMA-stained whole mouse lung was conducted using a light-sheet microscope (Ultra Microscope, LaVision BioTec, Germany) equipped with a 2× objective lens (MVPLAPO 2XC, NA 0.5, WD 10 mm) and a dipping cap. Thin planes of light illuminated the lung sampllige from both sides and merged images were acquired. Images were captured with a Neo sCMOS camera (Andor Technology) and saved as TIFF image stacks using ImSpector software (LaVision BioTec). For full-organ imaging, z-stacks with 5 μm intervals were acquired through light-sheet fluorescence microscopy (LSFM), and 3D reconstruction and rendering were performed using IMARIS software.

Supplementary Figures and Figure legends

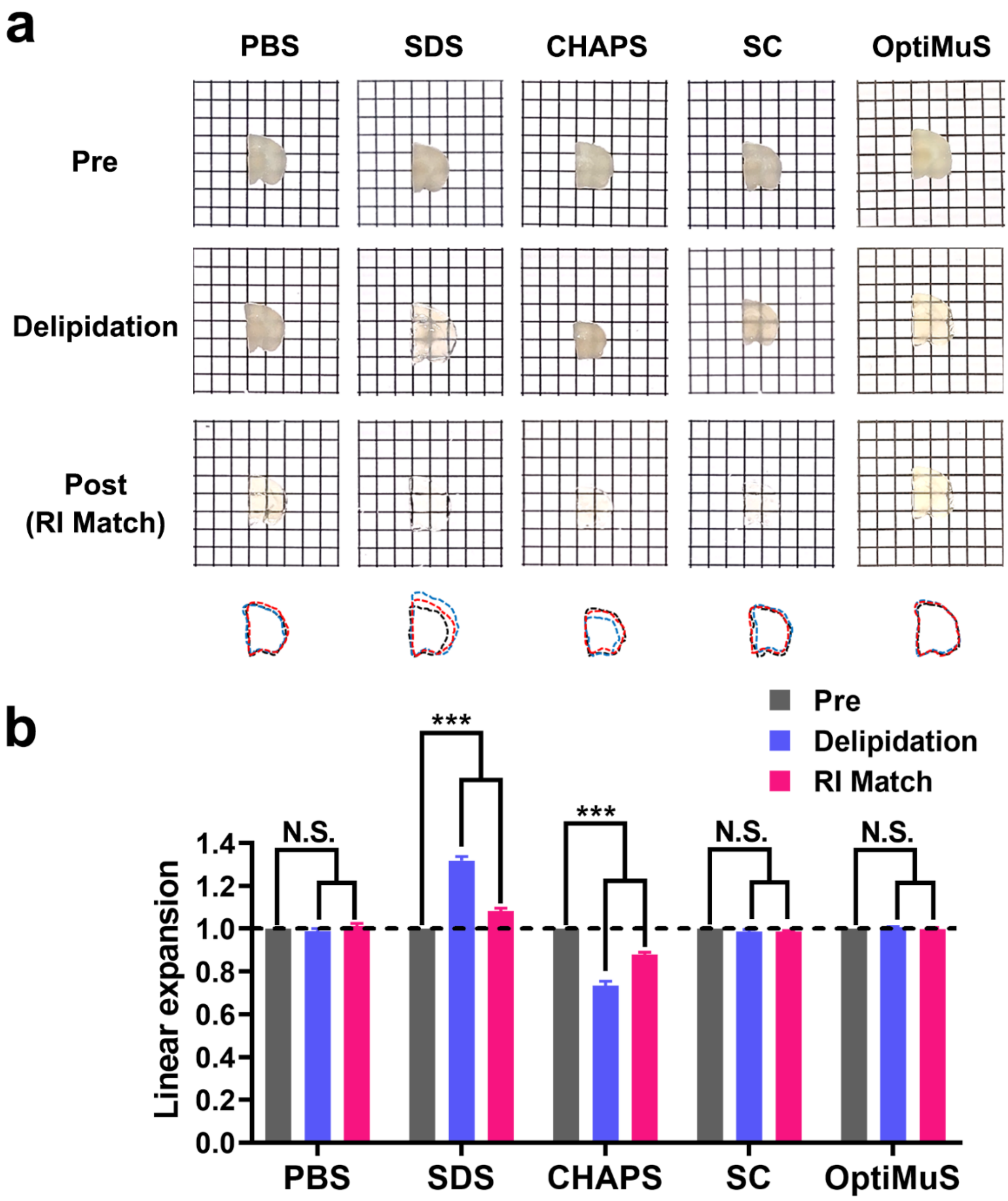

Supplementary Fig. 1. Comparative assessment of OptiMuS-*prime* versus conventional detergents for tissue clearing and expansion performance. (a) Bright-field images of a 1 mm-thick mouse brain slice before (Pre), after delipidation (Delipidation), and RI matching after clearing (Post) with various detergents. Overlapped outlines of pre-cleared (black), delipidated (blue), and post-cleared (red) brain tissues. Grid size = 2.5 mm x 2.5 mm. (b) Quantification comparison of linear expansion during the clearing process (n =3). Data are shown as the mean ± SD. *** $p<0.001$, ** $p<0.01$, * $p<0.05$.

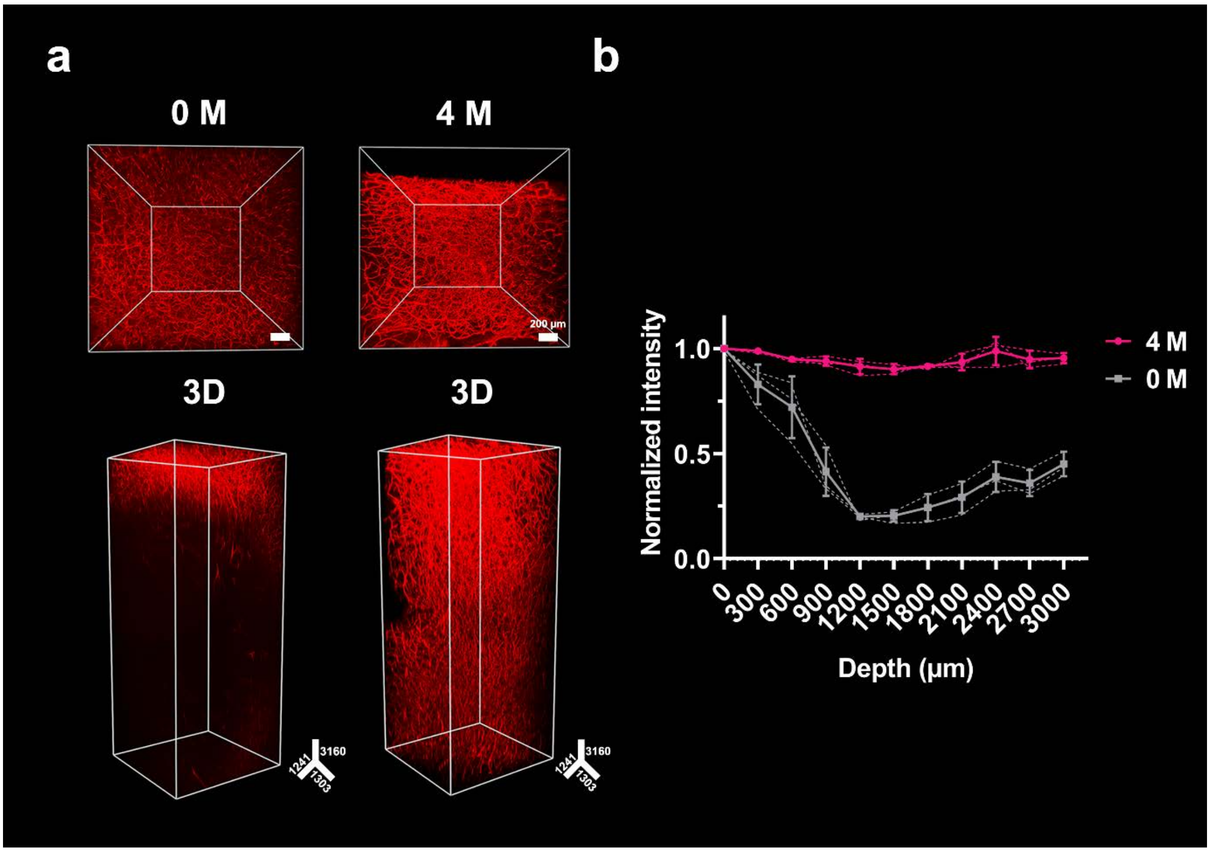

Supplementary Fig. 2. Urea enhances the delipidation capability of OptiMuS-*prime*. (a) Top view and 3D reconstruction images of a 3.2 mm-thick mouse brain stained with lectin dye after OptiMuS-*prime* processing: 0 M urea (left) 4 M urea (right). Scale bar = 200 μm. The 3D axis is expressed in μm. (b) Normalized fluorescence intensity curves of optical section images according to urea concentration and depth, normalized to intensity at 0 μm depth. Data were shown as the mean ± SD (n=3).

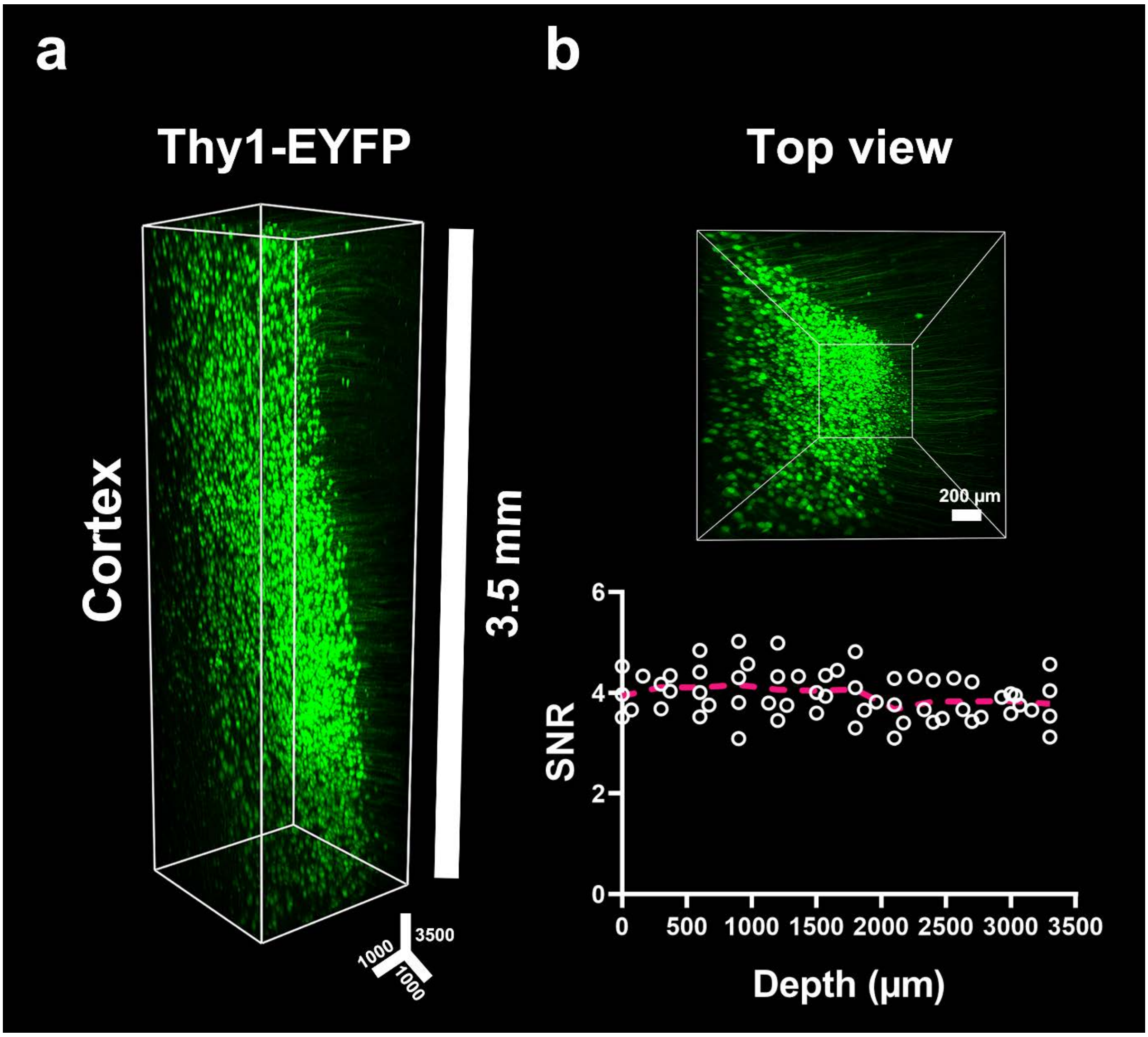

Supplementary Fig. 3. OptiMuS-*prime* preserves the fluorescence signal of endogenous proteins. (a) 3D reconstruction image of 3.5 mm- thick *Thy1*-EYFP transgenic brain sample after processing with OptiMuS-*prime*. The 3D axis is expressed in µm. (b) SNR values over the depth of imaging in (a) (n=3). Scale bar = 200 µm.

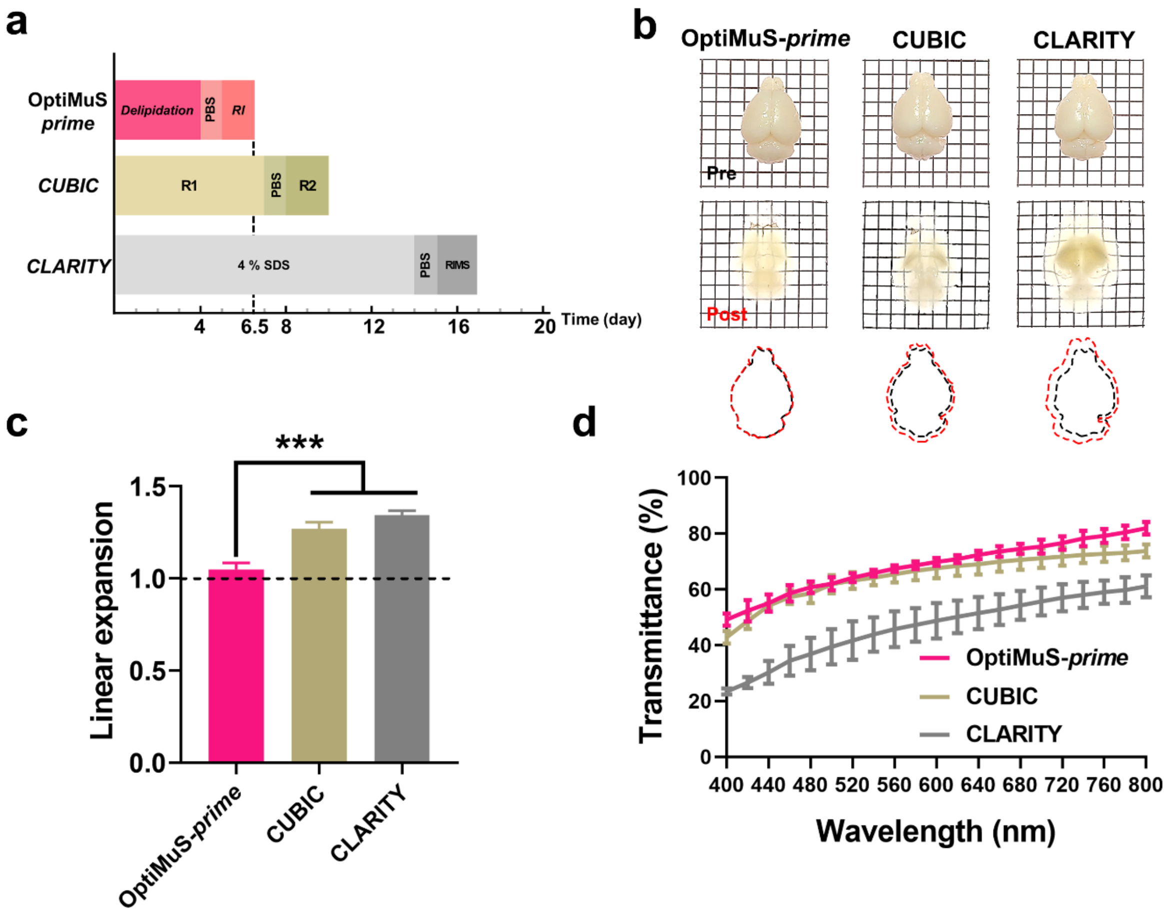

Supplementary Fig. 4. OptiMuS-*prime* showed excellent performance for whole mouse brain clearing, delivering a well-balanced combination of transparency, clearing efficiency, and structural preservation. (a) Timelines for clearing whole mouse brain samples using OptiMuS-*prime*, CUBIC, and CLARITY. (b) Bright-field images of whole mouse brains before and after clearing with each method in (a). Overlapped outlines of pre-cleared (black) and post-cleared (red) brain tissues show size changes. Grid size = 2.5 mm × 2.5 mm. (c) Quantitative comparison of linear expansion in brain tissues after each clearing method (n = 3). (d) Transmittance scan curves (400–800 nm) for whole mouse brain samples cleared with each method (n = 3). Data are presented as mean ± SD. ***p<0.001.

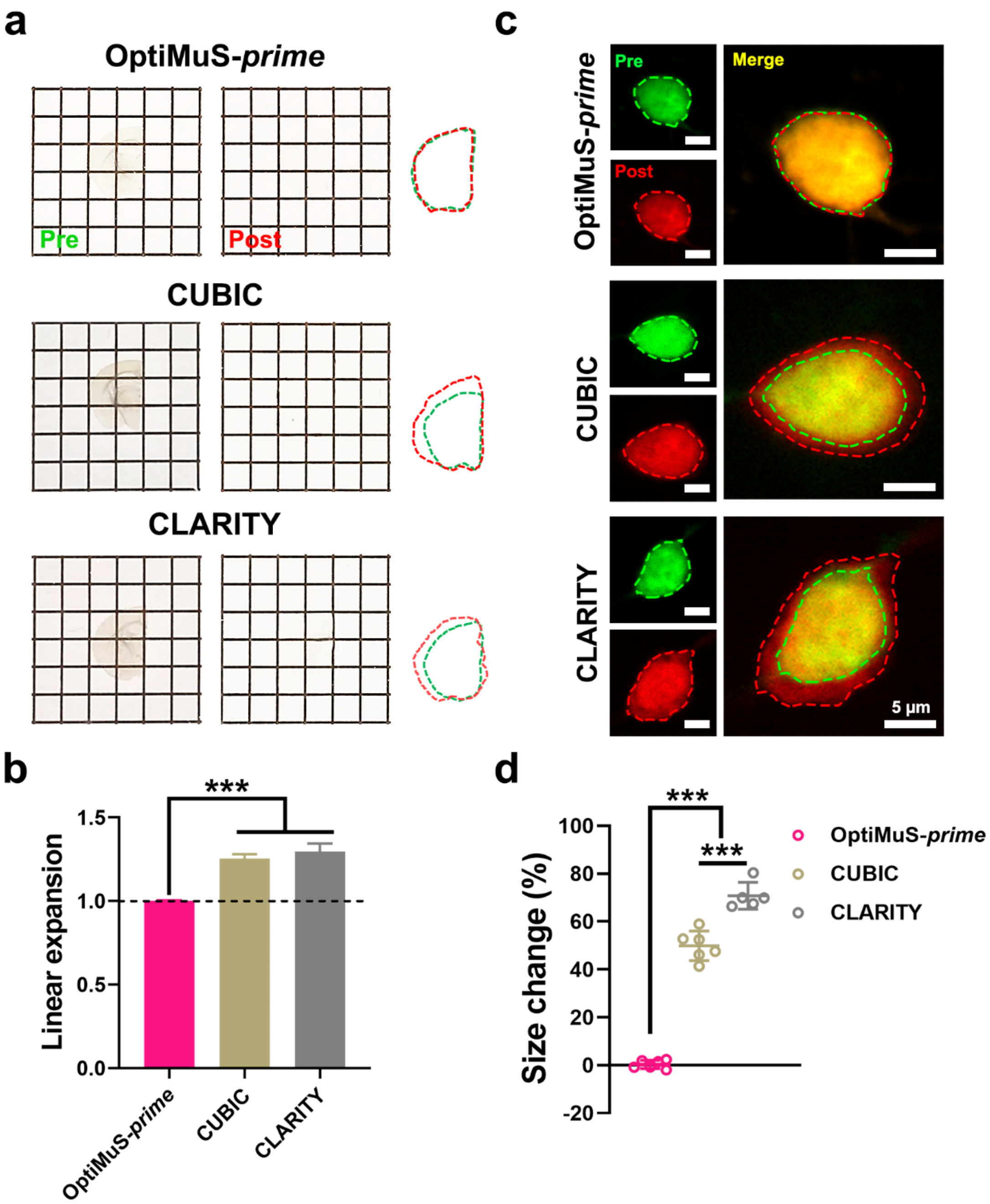

.

Supplementary Fig. 5. OptiMuS-*prime* exhibited superior preservation of brain tissue size and neuronal morphology at the cellular level compared to other clearing methods. (a) Bright-field images of 150 μm-thick ChAT-Cre::tdTomato brain slices before and after clearing with each method. Overlapped outlines of pre-cleared (green) and post-cleared (red) tissues highlight size changes. Grid size = 2.5 mm × 2.5 mm. (b) Quantitative comparison of linear expansion in brain tissues post-clearing with each method. (c) Fluorescence optical section images of 150 μm-thick ChAT-Cre::tdTomato brain slices

before and after clearing with each method, showing pre-cleared (top left), post-cleared (bottom left), and merged (right) images. Overlapped outlines of pre-cleared (green) and post-cleared (red) tissues highlight size changes. (d) Quantitative comparison of size changes in neuronal morphology areas post-clearing with each method. Data are presented as mean ± SD. ***$p<0.001$.

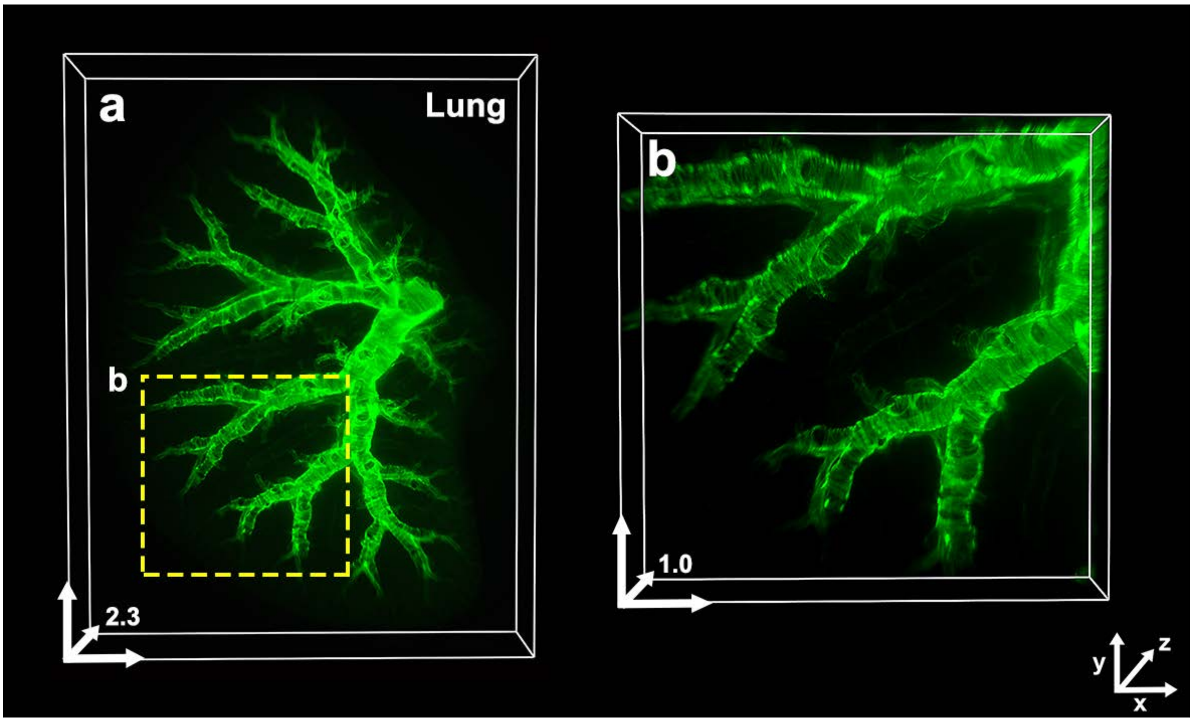

Supplementary Fig. 6. OptiMuS-*prime* enables visualization of the arteriolar architecture of the entire lung. (a) 3D view image of an α-SMA stained mouse whole lung processed with OptiMuS-*prime.* Detailed 3D reconstruction of the vasculature from the region highlighted in (a). The scale bars for the x and y axes in 3D coordinates of figures represent 2 mm (a), and 500 µm (b) while the z-axis indicates full tissue depth (mm).

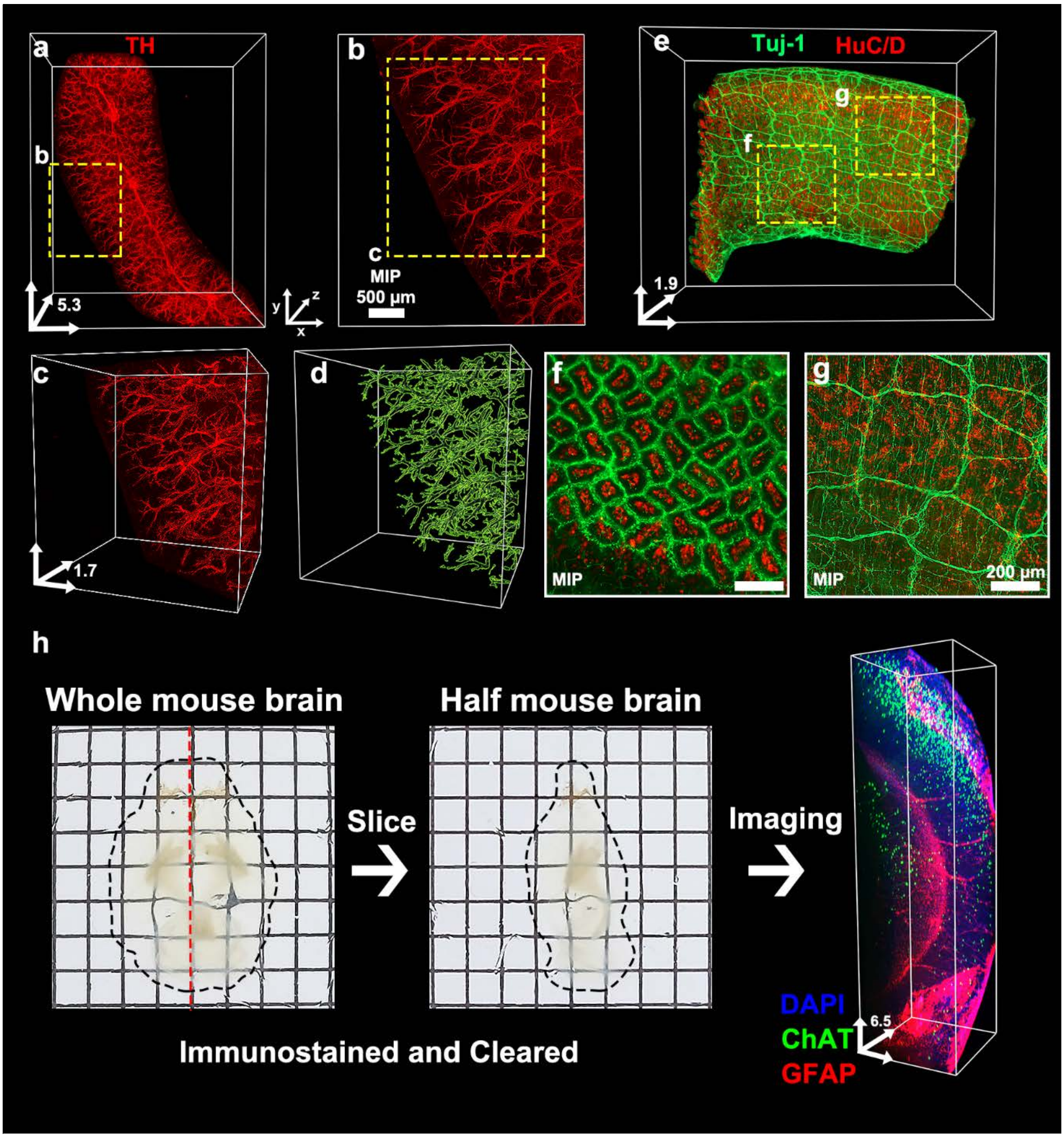

Supplementary Fig. 7. OptiMuS-*prime* enables efficient 3D visualization of neuronal markers in other mouse organs. (a) 3D reconstruction image of sympathetic nerve innervations visualized by anti-TH immunostaining in an intact mouse spleen processed using OptiMuS-*prime.* (b) Magnified MIP image of anti-TH-stained mouse spleen corresponding the region highlighted in (a). (c-d) Detailed 3D reconstruction(c) and 3D-rendered reconstruction image (d) of panicle-shaped anti-TH-stained mouse spleen corresponding the region highlighted in (b). (e) 3D reconstruction images of anti-Tuj-1 and anti-HuC/D co-stained mouse whole intestine; (green) Tuj-1, (red) HuC/D. (f) Magnified MIP

image of the mouse intestine crypt corresponding the region highlighted in (e). Scale bar = 200 µm. (g) Magnified MIP image of mouse intestine enteric nerves corresponding the region highlighted in (e). Scale bar = 200 µm. (h) Bright-field image of the intact whole mouse brain after OptiMuS-*prime* processing, immunostaining, and RI-matching (left); image of the halved brain (middle); and 3D volumetric reconstruction acquired using light-sheet fluorescence microscopy (right). Grid size = 2.5 mm × 2.5 mm. The scale bars for the x and y axes in 3D coordinates of figures represent 2 mm (a), 500 µm (c-e) while the z-axis indicates full tissue depth in mm (a, e, c, h) and µm (b).

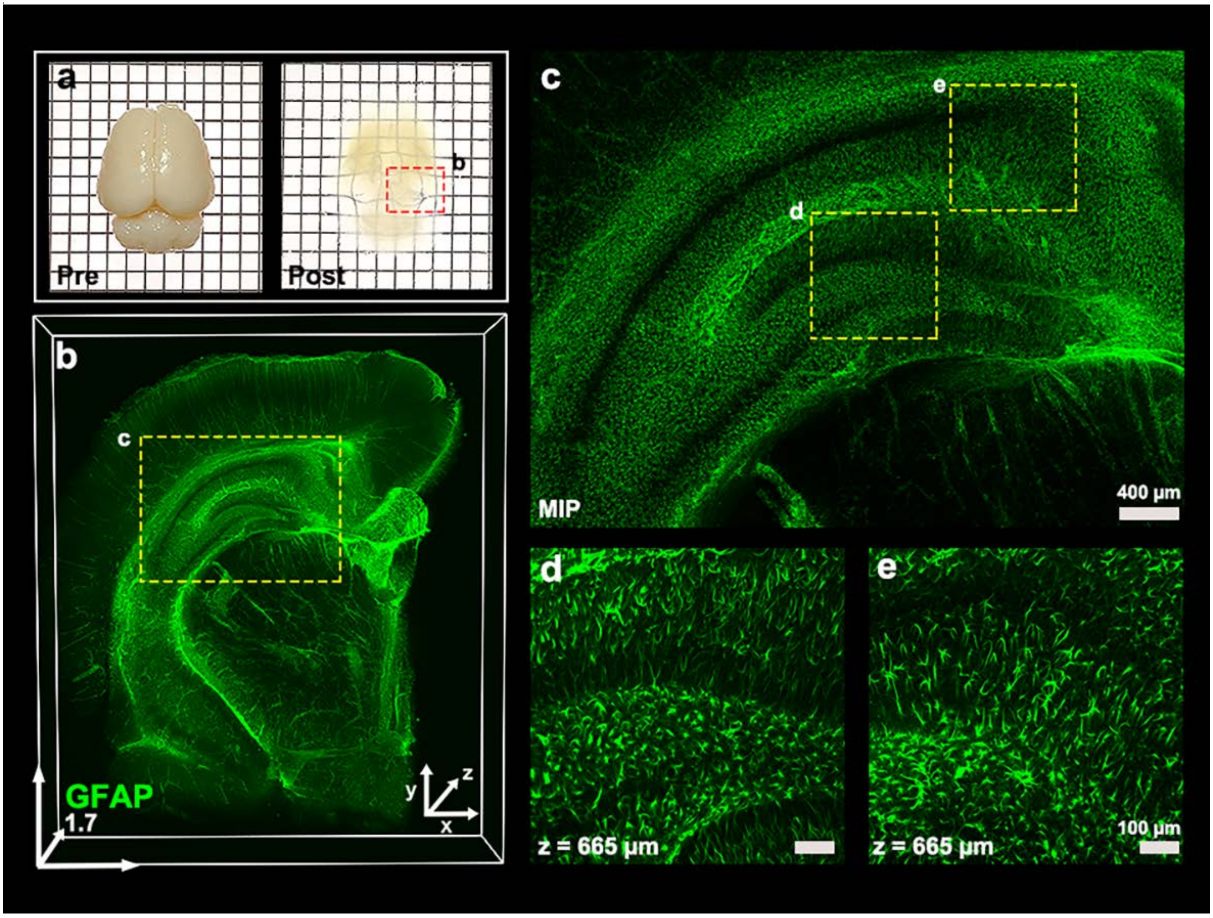

Supplementary Fig. 8. OptiMuS-*prime* facilitates scalable clearing of whole rat brain tissue. (a) Bright-field images of a whole rat brain before and after clearing with OptiMuS-*prime*. Grid size = 2.5 mm × 2.5 mm. (b) 3D view image of coronal sections from the whole rat brain in (a), cleared with OptiMuS-*prime* and stained with anti-GFAP antibody. The scale bars for the x and y axes in 3D coordinates represent 2 mm while the z-axis indicates full tissue depth in mm. (c) Magnified maximum intensity projection (MIP) image of the hippocampal region highlighted in (b). Scale bar = 400 μm. (d, e) Magnified optical section images of the area shown in (c). Scale bar = 100 μm.

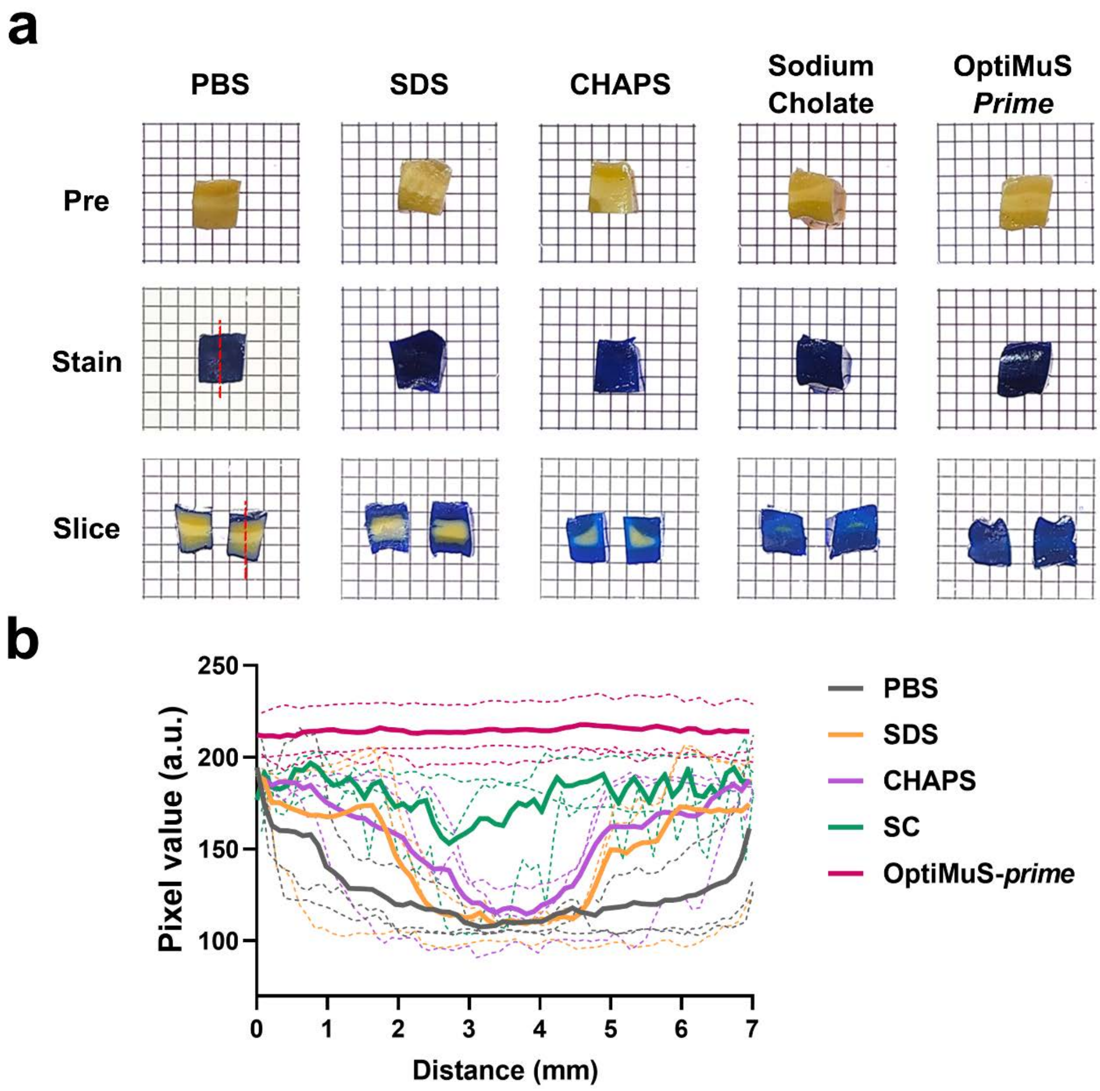

Supplementary Fig. 9. OptiMuS-*prime* demonstrates superior delipidation capability for postmortem human brain samples. (a) Bright-field images of 7-mm-thick human brain samples stained with Coomassie blue following delipidation with various detergents for efficiency evaluation. OptiMuS-*prime* demonstrates superior tissue permeabilization for dye penetration compared to SDS, CHAPS, and SC. Images show pre-delipidation (top), stained (middle), and sliced (bottom). The red dotted line indicates the sectioning plane. Grid size = 2.5 mm × 2.5 mm. (b) Intensity plot along the distance, derived from the red line on the slice image in (a). Data are presented as mean ± SD (n=3).

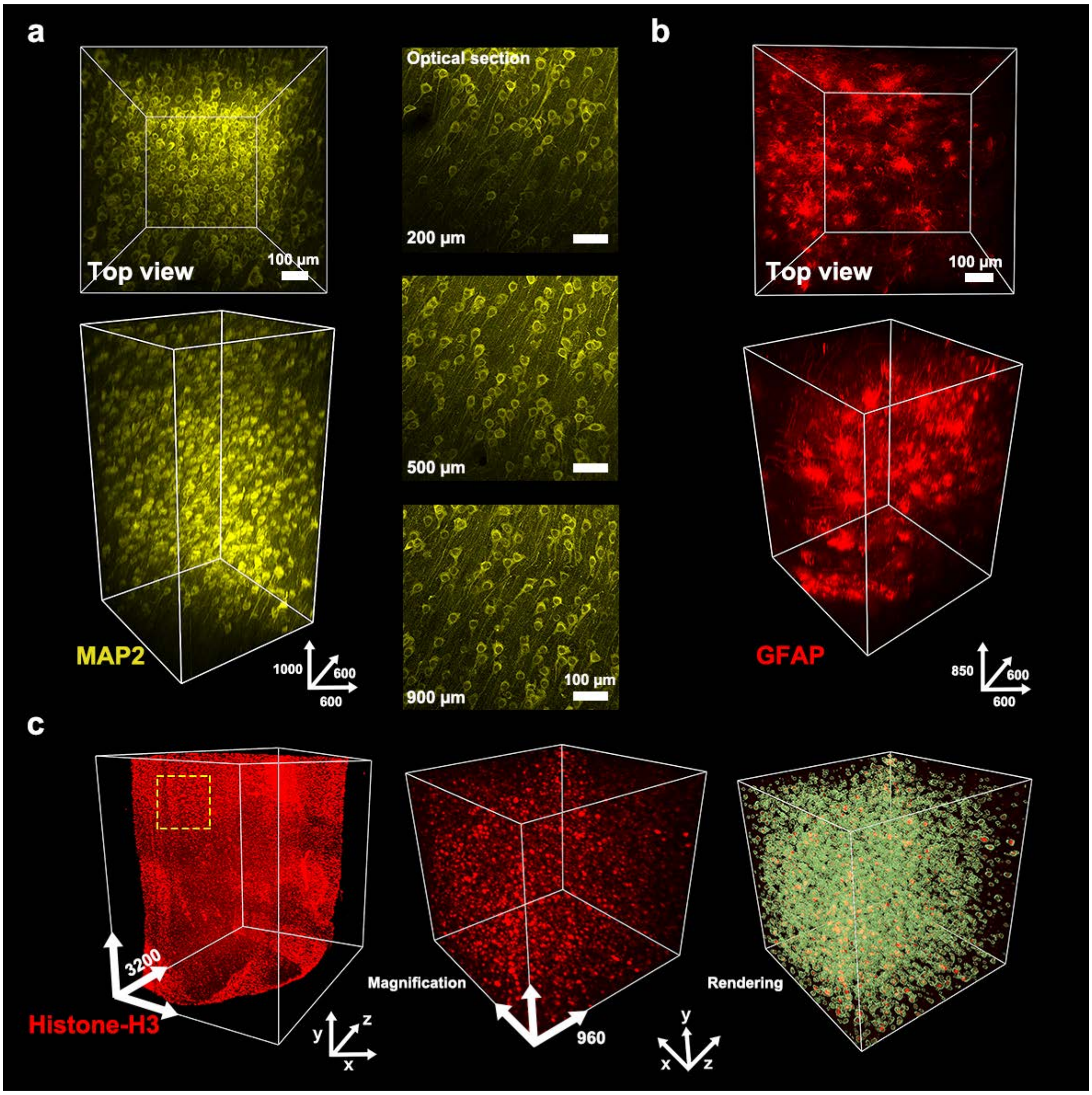

Supplementary Fig. 10. OptiMuS-*prime* enables high-resolution immunostaining of human brain tissue with various antibodies. (a) Top view and 3D reconstruction of a 1 mm-thick human brain sample stained with MAP2 after OptiMuS-*prime* processing, acquired by confocal microscopy. Left: overview; Right: optical sections at 200, 500, and 900 µm depths. (b) Top view and 3D reconstruction of a 1 mm-thick human brain sample stained with GFAP after OptiMuS-*prime* processing, acquired by confocal microscopy. The 3D axis is expressed in µm. (c) 3D reconstruction of a human brain block stained with Histone-H3 (nuclear marker) after OptiMuS-*prime* processing, acquired by light-sheet microscopy. Middle: detailed view; Right: 3D-rendered reconstruction. The scale bars for the x and y axes in 3D coordinates of figures represent 1mm (left), 200 µm (middle) while the z-axis indicates full tissue depth in µm.

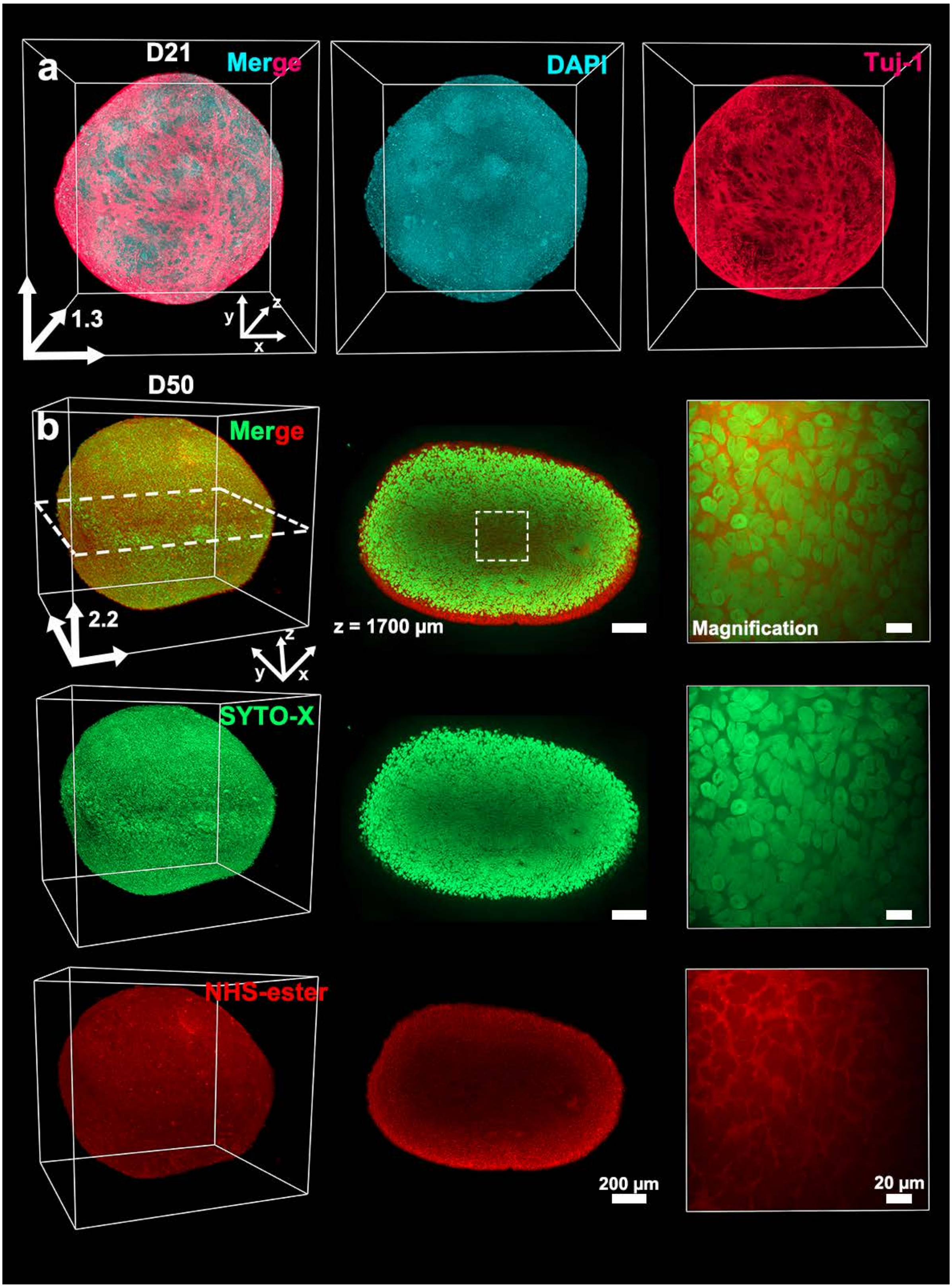

Supplementary Fig. 11. OptiMuS-*prime* enables 3D visualization of human brain organoids at various developmental stages. (a) 3D reconstruction image of D21

human brain organoids processed with OptiMuS-*prime* and stained with DAPI and Tuj-1. Left: merged image; Middle: DAPI; Right: Tuj-1. (b) 3D reconstruction images of D50 human brain organoid processed with OptiMuS-*prime* and stained with SYTO-X and NHS-ester dye. The central region was imaged at higher magnification using 40X confocal microscopy. Left: 3D image; Middle: optical section; Right: magnified view. Scale bars = 200 µm (Middle) and 20 µm (Right). The scale bars for the x and y axes in 3D coordinates of figures represent 500 µm while the z-axis indicates full tissue depth in mm.

Supplementary Tables.

Supplementary Table 1. Antibodies used in Experimental Procedures.

	**Antibody**	**Company**	**Host**	**Cat #**
**Primary Antibody**	Anti-GFAP antibody	Abcam	Goat	ab53554
	Monoclonal Anti-GFAP antibody (GA5), eFlour 660 conjugated	Invitrogen	Mouse	50-9892-82
	Monoclonal Anti-GFAP Antibody (GA5), Alexa Fluor 488 conjugated	Invitrogen	Mouse	53-9892-82
	Anti-alpha smooth muscle Actin antibody	Abcam	Rabbit	ab5694
	Monoclonal Anti-Alpha-Smooth Muscle Actin Antibody (1A4), Alexa Fluor 488 conjugated	Invitrogen	Mouse	53-9760-82
	Anti-MAP2 antibody	SYSY	Rabbit	188 002
	Anti-MAP2 antibody, Alexa Fluor 647 conjugated	Abcam	Rabbit	ab225315
	Monoclonal Anti-Neurofilament 200 (Phos. and Non-Phos.) antibody	Sigma aldrich	Mouse	N0142
	Anti-NeuN antibody	Sigma aldrich	Rabbit	ABN78
	Anti-Iba1 antibody	FUJIFILM Wako Chemicals	Rabbit	019-19741
	Anti-Iba1 antibody	Abcam	Rabbit	ab178847
	Anti-beta III Tubulin antibody	Abcam	Rabbit	ab18207
	Anti-Tyrosine Hydroxylase antibody	Abcam	Rabbit	ab112
	Monoclonal Anti-HuC/HuD Antibody	Invitrogen	Mouse	a21271
	Monoclonal Anti-Histone H3 (D1H2) XP antibody, Alexa Fluor 647 Conjugate	Cell Signaling Technology	Rabbit	12230S

	**Antibody**	**Company**	**Host**	**Cat #**
**Secondary Antibody**	Alexa Fluor 488, F(ab') 2-Goat anti-Rabbit IgG (H+L)	Invitrogen	Goat	A11070
	Alexa Fluor 568, Goat anti-Rabbit IgG (H+L)	Invitrogen	Goat	A11011
	Alexa Fluor 647, F(ab') 2-Goat anti-Rabbit IgG (H+L)	Invitrogen	Goat	A21246

	Alexa Fluor 594 Fab Fragment Goat Anti-Mouse IgG1	Jackson ImmuoResearch	Goat	115-587-185
	Alexa Fluor 647, F(ab') 2-Goat anti-mouse IgG (H+L)	Invitrogen	Goat	A21237
	Alexa Fluor 488 Donkey anti-Goat IgG (H+L)	Invitrogen	Donkey	A11055

Supplementary Table 2. Fluorescent probes used in Experimental Procedures.

	**Probe**	**Company**	**Cat #**
**Fluorescent Probes**	DyLight 594 labeled Lycopersicon Esculentum (Tomato) Lectin	Vector Labs	DL-1177
	SYTOX Green Nucleic Acid Stain	Invitrogen	S7020
	Atto 647N NHS ester	Sigma Aldrich	18373
	DAPI	Sigma Aldrich	D9542
